# Coregulation of Terpenoid Pathway Genes and Prediction of Isoprene Production in *Bacillus subtilis* Using Transcriptomics

**DOI:** 10.1371/journal.pone.0066104

**Published:** 2013-06-19

**Authors:** Becky M. Hess, Junfeng Xue, Lye Meng Markillie, Ronald C. Taylor, H. Steven Wiley, Birgitte K. Ahring, Bryan Linggi

**Affiliations:** 1 Bioproducts, Sciences and Engineering Laboratory, Washington State University Tri-Cities, Richland, Washington, United States of America; 2 Chemical and Biological Signature Sciences Group, Pacific Northwest National Laboratory, Richland, Washington, United States of America; 3 Fundamental and Computational Sciences, Pacific Northwest National Laboratory, Richland, Washington, United States of America; 4 Computational Biology and Bioinformatics Group, Pacific Northwest National Laboratory, Richland, Washington, United States of America; 5 Environmental and Molecular Sciences Laboratory, Pacific Northwest National Laboratory, Richland, Washington, United States of America; National Institutes of Health, United States of America

## Abstract

The isoprenoid pathway converts pyruvate to isoprene and related isoprenoid compounds in plants and some bacteria. Currently, this pathway is of great interest because of the critical role that isoprenoids play in basic cellular processes, as well as the industrial value of metabolites such as isoprene. Although the regulation of several pathway genes has been described, there is a paucity of information regarding system level regulation and control of the pathway. To address these limitations, we examined *Bacillus subtilis* grown under multiple conditions and determined the relationship between altered isoprene production and gene expression patterns. We found that with respect to the amount of isoprene produced, terpenoid genes fall into two distinct subsets with opposing correlations. The group whose expression levels positively correlated with isoprene production included *dxs*, which is responsible for the commitment step in the pathway, *ispD*, and two genes that participate in the mevalonate pathway, *yhfS* and *pksG*. The subset of terpenoid genes that inversely correlated with isoprene production included *ispH, ispF, hepS, uppS, ispE,* and *dxr*. A genome-wide partial least squares regression model was created to identify other genes or pathways that contribute to isoprene production. These analyses showed that a subset of 213 regulated genes was sufficient to create a predictive model of isoprene production under different conditions and showed correlations at the transcriptional level. We conclude that gene expression levels alone are sufficiently informative about the metabolic state of a cell that produces increased isoprene and can be used to build a model that accurately predicts production of this secondary metabolite across many simulated environmental conditions.

## Introduction

Isoprene (2-methyl-1,3-butadiene) is an important commodity due to its use as an aviation fuel and as a platform chemical for synthetic chemistry. Renewable methods for producing isoprene are being investigated to meet product demand and reduce the environmental impact of current production methods, which involve petroleum cracking [1], [2]. To this end, methods for large scale production of isoprene from a microbial host are being explored as cleaner sources of raw material [3]. End products of the isoprenoid pathway in bacteria are involved in a multitude of essential functions, such as protein degradation and hormone-based signaling. In addition, some are structural components of membranes, such as sterols, carotenoids, ubiquinone, and dolichols [3], [4]. Given the importance of these compounds in cell physiology and its utility as an industrial product, there is great interest in understanding the regulation of the enzymes that control the metabolism of these compounds.

Most animals, plants, and bacteria produce isoprene [5]–[7]. It is produced from one of two pathways: the mevalonate pathway, which is the predominant pathway in yeast and mammals, and the 1-deoxy-D-xylulose-5-phosphate (DXP) pathway, which is functional in most plants and microorganisms [6]. The role of isoprene in bacteria has not been firmly established [8]–[10], but the toxicity of isoprene precursors and the requirement of isoprenoid compounds for normal growth strongly suggest that regulation of this pathway is tightly controlled by the host cell, and must be considered in efforts to alter isoprene production levels in model or non-model organisms [8], [11]. Collectively, we refer to the 11 genes of the DXP pathway and three genes of the mevalonate pathway as the terpenoid genes. One potential way to control the expression of the enzymes in this pathway is through operon usage or shared transcription factors as part of one or more regulons [12]–[18]. These regulatory controls are common in bacteria, but have not yet been described for the members of the DXP pathway [8].

The enzymatic cascade responsible for isoprene and isoprenoid production includes 11 gene products [19], [20]. The *dxs* gene encodes the first enzyme in the pathway that condenses pyruvate and glyceradehyde-3-phosphate to generate 1-deoxy-D-xyulose-5-phosphate. The next six steps in the pathway, encoded by *dxr*, *ispD*, *ispE* (also referred to as *ipk*), *ispF*, *ispG,* and *ispH* are required to produce both of the five carbon prenyl diphosphates, dimethylallyl diphosphate (DMAPP), and isopentenyl diphosphate (IPP). The Idi enzyme (isopentenyl pyrophosphate isomerase), encoded by the *fni* gene, is responsible for the isomerization reaction between DMAPP and IPP. Evidence from previous studies suggests that DMAPP is converted to isoprene by isoprene synthase (IspS), an enzyme identified in plants but currently unidentified in bacteria [7], [8], [11], [21]. Farnesyl diphosphate synthase (IspA) condenses DMAPP and IPP to produce geranyl diphosphate (GPP), farnesyl diphosphate (FPP), and larger order isoprenoid compounds. Both heptaprenyl diphosphate synthase (HepS) and undecaprenyl pyrophosphate synthetase (UppS) are downstream of IspA in the pathway and also synthesize larger order isoprenoids.

The genes *yhfS*, *mmgA*, and *pksG* are expressed in *B. subtilis* but are annotated to be part of a dead-end mevalonate pathway [22], [23]. However, it has been suggested that polyketide biosynthesis is linked to isoprenoid biosynthesis in *B. subtilis* [24]. Furthermore, the activities of pathways that regulate the metabolic pool available for the terpenoid pathway, such as pyruvate, are likely to influence isoprene production. Attempts to produce significant amounts of isoprene in engineered bacteria would benefit from a mechanistic understanding of the how the terpenoid genes are regulated in the native host, thereby facilitating synthetic biology approaches to control isoprene production levels in engineered microbes [18], [25], [26].

We hypothesized that the RNA expression levels of genes that modulate isoprene production are strictly controlled and regulated under environmental conditions that increase isoprene production. Accumulation of DMAPP and IPP is cytotoxic in *E. coli,* and to a lesser extent *B. subtilis* [11], [27], which is consistent with the idea that the genes underlying isoprene production are regulated. Thus, the expression levels of genes in the terpenoid pathway should correlate with the level of isoprene produced. To test this hypothesis, RNA was isolated from *B. subtilis* after being subjected to 12 different perturbations and the pattern of gene expression was determined using RNA-seq. We found that different subsets of the terpenoid genes were coregulated. We also produced a multivariate model using partial least squares regression (PLSR) analyses with a subset of 213 genes. The model was tested against 19 different conditions from an independent experiment and accurately predicted isoprene production levels from these perturbations. Therefore, the gene expression-based model can be used to identify the conditions and pathways that cooperate to maximize isoprene production.

## Materials and Methods

### Bacterial Strains and Growth Conditions

*Bacillus subtilis* strain DSM10 (ATCC 6051) was obtained from the German Collection of Microorganisms and Cell Cultures and grown in Luria-Bertani (LB, Fisher Scientific) broth at 30°C with continuous shaking. The *E. coli* strain DH5α (Invitrogen), used in *B. subtilis* cloning efforts, was grown in LB media at 37°C at 180 rpm. When required, the culture media was supplemented with antibiotics (ampicillin, 100 µg/mL and chloramphenicol, 10 µg/mL, Fisher Scientific).

### Cloning and Sequencing of*dxs, dxr, fni*, and *ispA* Genes

The *dxs, dxr, fni,* and *ispA* genes were PCR-amplified from *B. subtilis* DSM10 genomic DNA and cloned using the *B. subtilis* – *E. coli* shuttle vector pHT01 (MoBiTec) [28] according to the method used by Xue et al. [3]. The recombinant plasmids are referred to as OX-*dxs*, OX-*dxsdxr*, OX-*dxsfni*, OX-*fni*, and OX-*ispA*. The empty pHT01 vector was also transformed into *B. subtilis* using the same method, and is referred to in the text as empty vector. A list of primers used in the study is provided in Table S1.

### Perturbations

A starter culture of DSM10 (in 10 mL LB media) was inoculated with a single colony and grown overnight (16 to 20 hr) at 30°C with shaking at 180 rpm. The optical density of cultures at 600 nm (OD_600_) was determined using a JENWAY 6405 UV/VIS Spectrophotometer. The starter culture was diluted to an OD_600_ of 0.05 using fresh LB media to a total volume of 100 mL. When cultures reached an OD_600_ of 0.5, 10 mL aliquots of the culture was transferred to 20 mL GC-MS sampling vials. At this point, the indicated treatment was applied (two vials per treatment, biological replicates were independent from this point forward). Genetic perturbations by overexpression of genes in the mutant strains were induced at mid-log phase by the addition of 1 mM isopropyl β-D-1-thiogalactopyranoside (IPTG, Fisher Scientific). Environmental perturbations were conducted as follows: wild type control, no additions; acetic acid (glacial acetic acid, Fisher Scientific), 0.2% or 2%; ethanol (200 proof molecular biology grade, Fisher Scientific), 0.1% or 1%; lactic acid (DL-Lactic Acid, Fisher Scientific), 0.2% or 2%; indole (Sigma-Aldrich, stock solutions prepared in molecular biology grade ethanol) 0.02 mg/mL or 0.2 mg/mL; hydrogen peroxide (Fisher Scientific), 0.005% or 0.02%; sodium chloride (Fisher Scientific), 0.3 M; glucose (Fisher Scientific), 0.2%; xylose (Fisher Scientific) 0.2%; mannose (Fisher Scientific) 0.2%; and dimethyl sulfoxide (DMSO; Fisher Scientific), 70 mM. The vials were tightly capped and incubated for three hours at 30°C with shaking at 150 rpm. After three hours, the culture density was determined (from one sample vial) and the concentration of isoprene in the headspace was measured (from the remaining sample vial) using the GC-MS method described below.

#### Detection of isoprene production using gas chromatography-mass spectrometry

We measured isoprene as described previously [3]. Briefly, the concentration of isoprene in the headspace of liquid cultures was detected using a gas chromatography-mass spectrometry (GC-MS) method. We used an Agilent 7890A GC and a 5975 mass selective detector (MSD) coupled with a CTC PAL autosampling system that was equipped with a solid-phase microextraction (SPME) syringe (Supelco) to absorb volatile compounds in the headspace. The fiber material was 50/30 divinylbenzene-carburen on polydimethylsiloxane on a stable flex fiber. A DB-5 ms column (length, 30 m; inner diameter, 0.25 mm; film thickness, 0.25 µm) with helium as the carrier gas at a flow rate of 0.9 mL/min was used. The oven temperature was initiated at 30°C for 3.5 min, and was increased at a rate of 20°C per min until 120°C. The headspace of LB media was used as a negative control. Bacterial isoprene production was identified by comparing peak retention times and the mass spectra profiles with an isoprene standard (Sigma-Aldrich). Calibration standards were prepared by adding various concentrations to 10 mL of LB media. Isoprene eluted at 1.9 min, and the isoprene concentrations produced in the bacterial cultures were calculated by conversion of the GC-MS peak area to nanograms of isoprene using the calibration curve.

### RNA Extraction

Bacterial cultures were centrifuged at 5,000× g for 10 minutes at room temperature, and the supernatant was discarded. RNA was extracted from the resulting cell pellet using the RiboPure™-Bacteria Kit (Life Technologies, AM1925). Briefly, 350 µL of ice-cold RNA_WIZ_ was added to the cell pellet and resuspended by vigorous vortexing. We note that the level of RNA species that are rapidly degraded may be altered during this short period between culturing and RNA stabilization and extraction (approximately 10 minutes). Cells were disrupted by beating in the presence of 250 µL of Zirconia beads for 10 minutes at room temperature. The resulting lysates were collecting after centrifuging for 5 min at 4°C. Lysates were frozen at −80°C for at least 12 hours prior to final RNA extraction. Upon removal from the freezer, 0.2 volumes of ice-cold chloroform was immediately added and pipetted vigorously until the lysate thawed. All subsequent protocol steps were conducted according to the manufacturer’s instructions, including use of the DNase treatment supplied with the kit. Extracted RNA was quantified using the NanoDrop ND-1000 (Fisher Scientific). RNA Integrity was determined using 1.0 µL of RNA sample with the Agilent RNA 6000 Nano Kit and chip (Agilent, part number 5067-1548) according to the manufacturer’s instructions with the Agilent 2100 BioAnalyzer. Samples were analyzed according to the total prokaryote RNA method software supplied with the Agilent 2100 BioAnalyzer instrument. Samples with a RIN score >7 were used for RNA-Sequencing.

### RNA-sequencing

The Applied Biosystems SOLiD™ Total RNA-Seq kit (catalog number 4445374) was used to generate the cDNA template library. The SOLiD™ EZ Bead system was used to perform emulsion clonal bead amplification to generate bead templates for SOLiD™ platform sequencing. Samples were sequenced on either the SOLiD™ 5500xl platform or the SOLiD™ 4 platform. The samples sequenced on the SOLiD™ 5500xl platform consisted of the two biological replicates (cultures supplemented independently). The training data set consisted of the following twelve samples: wild type control, 2% acetic acid, 1% ethanol, 2% lactic acid, 0.2 mg/mL indole, 0.005% and 0.02% H_2_0_2_, OX-*dxs*, OX-*fni*, 70 mM DMSO, empty vector control, and OX-*ispA*. One biological replicate for the OX-*fni* perturbation did not pass quality control metrics and was eliminated from further analysis, resulting in a single replicate for this condition. The samples sequenced on the SOLiD™ 4 platform consisted of the samples used in the testing data set with one replicate of each perturbation sequenced. The testing data set consisted of the following 19 samples: wild type control, 0.2% acetic acid, 2% acetic acid, 0.1% ethanol, 1% ethanol, 0.2% lactic acid, 2% lactic acid, 0.02 mg/mL indole, 0.2 mg/mL indole, 0.005% and 0.02% H_2_0_2_, 0.3 M NaCl, 0.2% glucose, 0.2% mannose, 0.2% xylose, OX-*dxs*, OX-*dxsdxr*, OX-*dxsfni*, and OX-*fni*. The 50-base short read sequences produced by the SOLiD™ 4 sequencer were mapped in color space using SOLiD™ BioScope™ software version 1.3. The 50-base short read sequences produced by the SOLiD™ 5500xl sequencer were mapped in color space using LifeTechnologies LifeScope™ software version 2.5.1. The same mapping algorithm was used for both sequencing platforms with the default settings. BioScope™ and LifeScope™ were instructed to map the short reads onto a reference genome and set of called genes from *B. subtilis* subsp. str. 168, a neighboring *B. subtilis* strain that has previously been sequenced (RefSeq NC_000964). BioScope™ and LifeScope™ were given a filtering file to use that included the 16S, 5S, and 23S rRNA sequences for *B. subtilis* (in addition to the adapter and barcode sequences to filter before mapping). Therefore, ribosomal filtering was done computationally, with BioScope™ and LifeScope™ discarding any reads matching any subsequence of the three rRNA sequences in the filter file. Each BioScope™ and LifeScope™ run produced a BAM file containing the sequence of every mapped read and its mapped location. The resulting BAM files were imported into AvadisNGS (version 1.3, Strand Sciences). BAM files are available in the Sequence Read Archive at http://www.ncbi.nlm.nih.gov/sra.

### Data Analyses

The expression values for each gene were quantitated using DE-Seq [29] normalization in Avadis NGS with the threshold set to one without mean centering and log_2_ transformed. The mean and standard deviation of each gene’s expression were calculated from the normalized expression using Matlab (2012a, Mathworks). The coefficient of variation (CV) for expression was <22% for at least 75% of the genes for the training dataset (2 biological replicates) and <33% for 75% of the genes when considering all three biological replicates (training and test dataset). We did not observe a perturbation-specific bias in CV in these experiments. Data used for clustering and modeling are included in Table S2. Clustering analyses were performed using the Matlab functions ‘clustergram’ and ‘kmeans’ as indicated with default parameters. Spearman’s rank correlation coefficients were calculated using the Matlab function ‘corr’ with the option “’type’, ‘Spearman’”. The reduced list of regulated genes (199 genes) was generated using analysis of variance (ANOVA) in AvadisNGS to select genes differentially expressed between the control and any other perturbation with a p<0.001 with Benjamini-Hochberg correction. Gene Ontology (GO) enrichment analyses were performed using DAVID Bioinformatics Resources [30].

Partial least squares regression analyses were performed using the Matlab toolbox ‘PLStoolbox’ (Eigenvector Research, Inc.) using the SIMPLS algorithm. Training data were mean-centered and auto-scaled to create a model with calibration fit of R^2^ = 0.99, a cross-validation fit (venetian blinds method with 6 splits) of R^2^ = 0.49, and the model predicted the response variable (isoprene production) in the test set with a fit R^2^ = 0.64. Models with higher prediction power were created when test data included the H_2_0_2_ perturbations; however, these perturbations created models that were highly dependent on these samples and were therefore removed to create a model more representative of the other perturbations.

## Results

### Identification of Perturbations that Alter Isoprene Production

**Figure 1:**
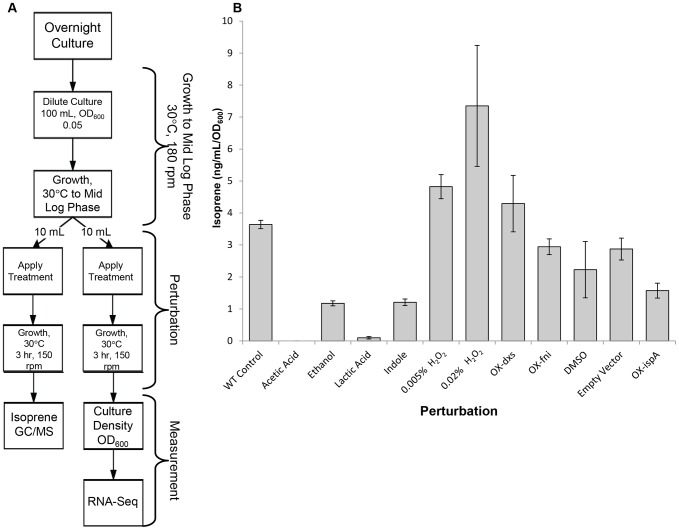
Quantitation of isoprene production under diverse experimental perturbations in*B. subtilis*. Flow chart of the perturbation and sampling protocols. Details are provided in Materials and Methods. B**,** The isoprene concentrations in the headspace were determined by GC-MS. “OX” represents genetic perturbations in which the listed gene was overexpressed. Bars indicate the mean and error bars indicate standard deviation from the mean.

The rate of isoprene production in *B. subtilis* is dependent on growth conditions and nutrient availability [8], [21]. To identify changes in gene expression that correspond to altered isoprene production, we screened for conditions that either increase or decrease isoprene production in *B. subtilis* strain DSM10. After media was supplemented with chemical stresses or nutrients, isoprene production in the culture head space was measured as previously described [3]. Because isoprene production levels correlate to both total cell number and phase of growth [3], [8], supplements were added mid-log phase (corresponding to an OD_600_ of 0.5) and isoprene levels were normalized to culture density (Figure 1A).

We identified eight conditions that altered the rate of isoprene production in wild type *B. subtilis*. Of these, the addition of acetic acid and lactic acid had the most dramatic effects on isoprene production and reduced it to nearly undetectable levels (Figure 1B). Ethanol, which induces chemical stress, or indole, which functions as a signaling molecule in bacteria and is required for amino acid biosynthesis [31], [32], reduced isoprene production, albeit to a lesser extent than either of the acids. Addition of DMSO, which negatively affects NAD^+^ synthetase enzymatic activity and alters the available NAD/NADH pool [33], [34], moderately but reproducibly lowered isoprene production. Hydrogen peroxide (H_2_O_2_), which induces oxidative stress, was the strongest inducer of isoprene production. In plants, it has been proposed that H_2_O_2_-induced isoprene production evolved as a mechanism to react with and mitigate the damaging effects of reactive oxygen species [35]–[38]. In total, we identified seven media supplements that altered the production of isoprene in *B. subtilis*: 2% acetic acid, 2% lactic acid, 0.2 mg/mL indole, 70 mM DMSO, 1% ethanol, and 0.005% and 0.02% H_2_0_2_.

**Figure 2:**
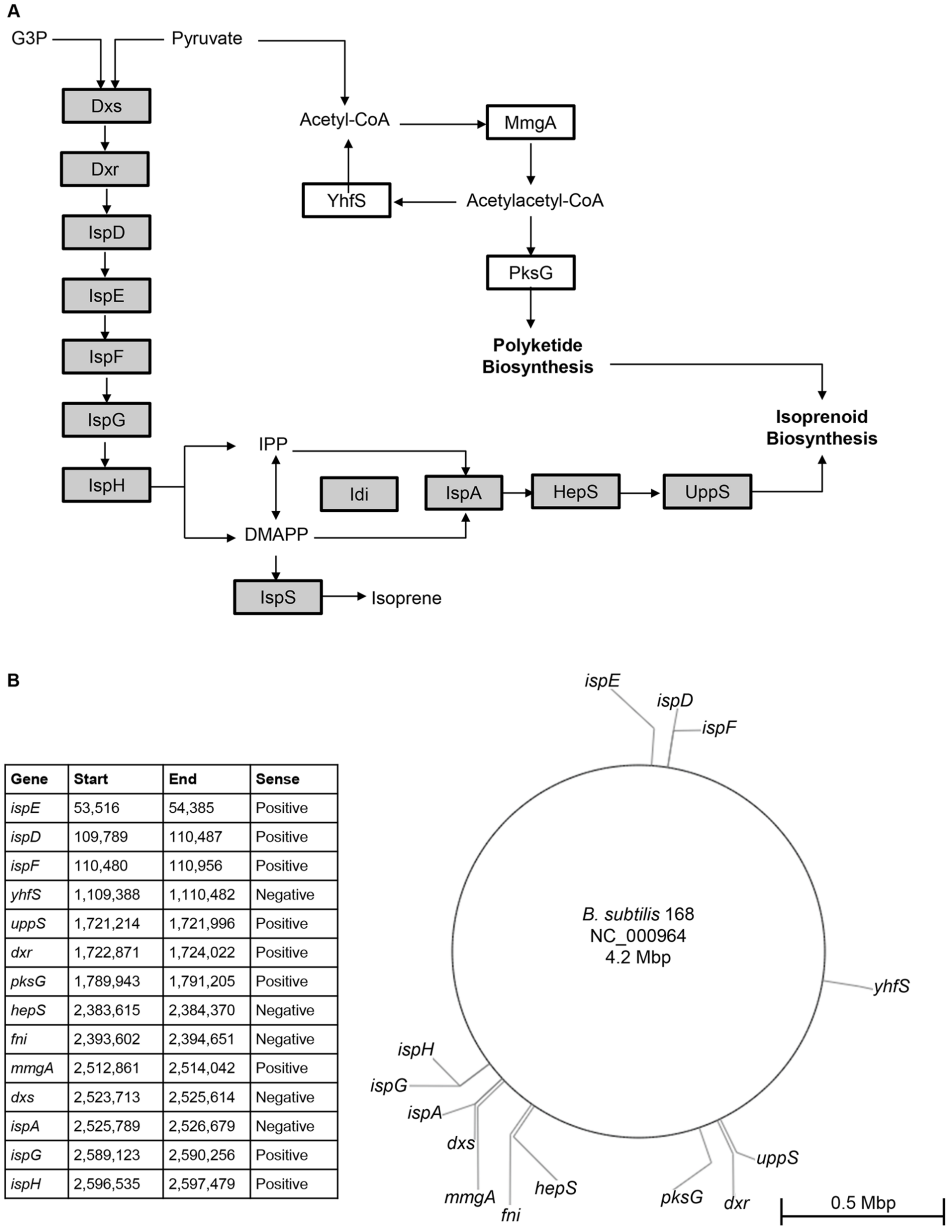
Terpenoid pathway genes in*B. subtilis*. Metabolic pathway layout of terpenoid genes. The 1-deoxy-D-xylulose-5-phosphate (DXP) pathway enzymes (shaded) are: Dxs, 1-Deoxy-D-xylulose-5-phosphate synthase; Dxr, 1-deoxy-D-xylulose-5-phosphate reductoisomerase; IspD, 4-diphosphocytidyl-2-C-methyl-D-erythritol synthase; IspE, 4-diphosphocytidyl-2-C-methyl-D-erythritol kinase; IspF, 2C-methyl-D-erythritol 2,4-cyclodiphosphate synthase; IspG, 1-hydroxy-2-methyl-2-(E)-butenyl 4-diphosphate synthase; IspH, 1-hydroxy-2-methyl-butenyl 4-diphosphate reductase; Idi (encoded by *fni*), isopentenyl pyrophosphate isomerase; IspA, farnesyl diphosphate synthase; HepS, heptaprenyl diphosphate synthase component I; UppS, undecaprenyl pyrophosphate synthetase; IspS, putative isoprene synthase. The mevalonate (MVA) pathway enzymes (white) are: MmgA, degradative acetoacetyl-CoA thiolase; YhfS, hydroxymethylglutaryl CoA synthase; PksG, 3-hydroxy-3-methylglutaryl-ACP synthase. Metabolite abbreviations: G3P, glyceraldehyde-3-phosphate; IPP, isopentenyl diphosphate; DMAPP, dimethylallyl diphosphate.B, The genome position, length, and sense of each of the terpenoid genes in the *B. subtilis* genome are represented in the table and graphically.

Genetic perturbations were also tested to determine if overexpression of selected enzymes in the DXP pathway would influence isoprene production. Isoprene production proceeds via the condensation of pyruvate and glyceraldehyde-3-phosphate by the enzyme Dxs into a metabolic cascade ultimately producing either isoprene or larger terpenoid compounds (Figure 2A) [8], [20], [22], [39]. Several enzymes in this pathway could serve as rate limiting steps in isoprene production. Specifically, metabolic flux through the DXP pathway appears to be heavily dependent on the activity of Dxs, Idi (encoded by *fni*), and IspA enzymes [9], [10], [40]–[42]. The regulation of these enzymes in bacteria, however, has yet to be fully described. These genes are not part of a single operon, therefore suggesting a more complicated regulatory strategy for these genes as well as for the entire terpenoid pathway (Figure 2B) [20], [22], [23]. To evaluate the influence of these enzymes on isoprene production, we overexpressed the genes encoding Dxs, Idi, and IspA. We found that *dxs* overexpression slightly increased isoprene levels, as described previously (Figure 1B) [43]–[45]. Although the Idi enzyme catalyzes the conversion of IPP to DMAPP (the substrate for IspS conversion of DMAPP to isoprene), our experiments showed that *fni* overexpression did not affect isoprene production levels, suggesting that conversion of IPP to DMAPP is not a rate-limiting step in isoprene production in LB growth media. Overexpression of *ispA*, which catalyzes the conversion of DMAPP and IPP to larger terpenoids (e.g., GPP and FPP), significantly reduces the level of isoprene production (Figure 1B), suggesting that *ispA* reduces the level of DMAPP, resulting in reduced substrate availability for IspS to convert to isoprene. In total, we identified 12 unique chemical and genetic perturbations that modulate isoprene production.

### Terpenoid Gene Clustering to Identify Patterns of Coregulation

To determine whether the expression of genes that modulate isoprene production are coordinately regulated, we isolated RNA from *B. subtilis* cultures that had been perturbed, and assessed the cellular transcription profile of two biological replicates for each perturbation using RNA-seq. We were particularly interested in the expression profile of genes involved directly in the DXP pathway, as well as *mmgA*, *yhfS*, and *pksG*, which are reported to belong to a “dead-end” mevalonate pathway in *B. subtilis* [22], [23]. The *pksG* gene is also known to play a functional role in polyketide biosynthesis, which has recently been linked to isoprenoid biosynthesis [24]. We collectively refer to these 14 genes as the terpenoid genes.

**Figure 3:**
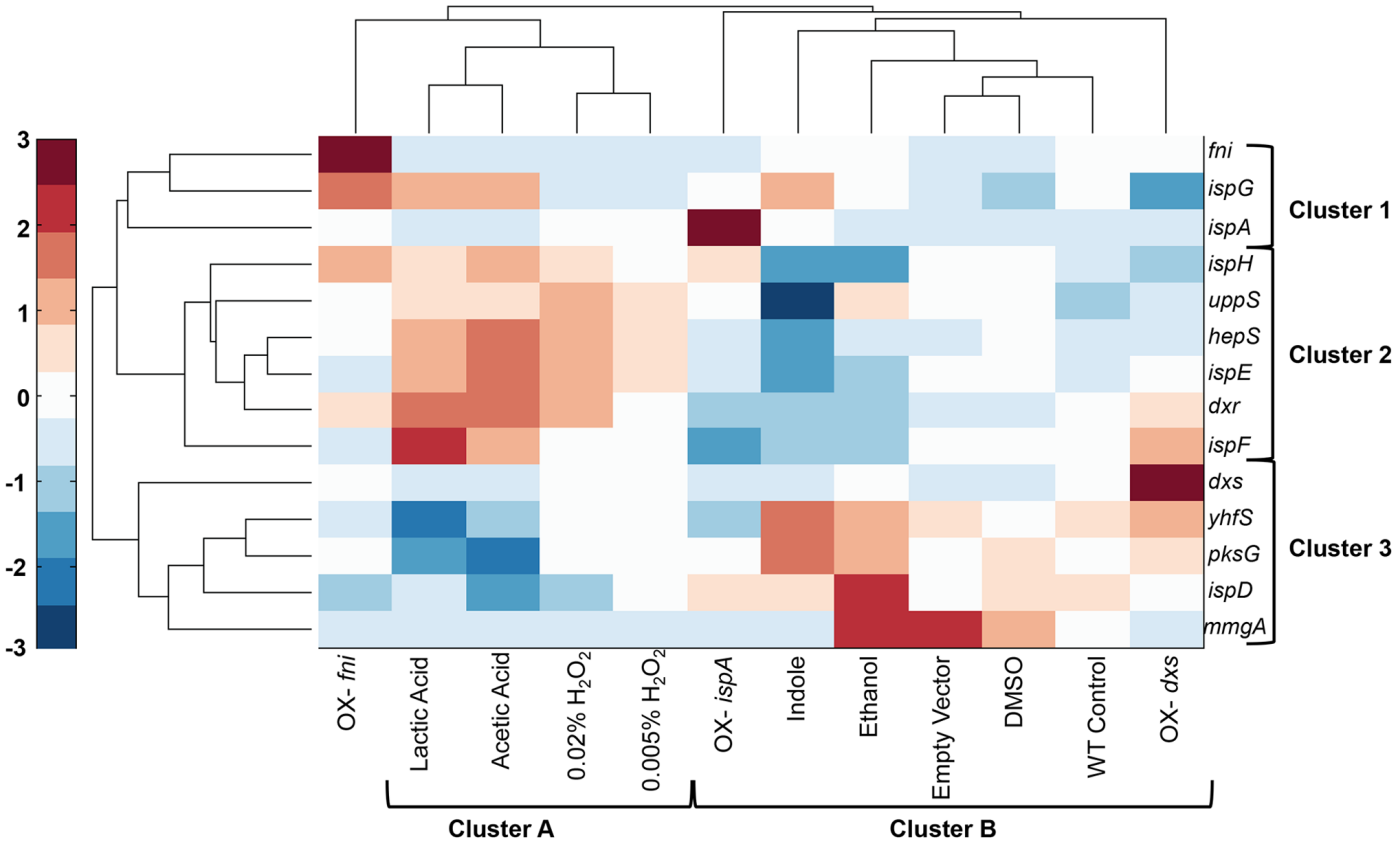
The terpenoid genes cluster into three main groups with respect to coexpression and into two main groups with respect to perturbations. Two-dimensional hierarchical clustering of the terpenoid genes against isoprene production in the investigated environmental and genetic perturbations in *B. subtilis*.

Hierarchical clustering was used to identify the terpenoid genes that are coexpressed under the 12 perturbations. The 14 genes are split into three main clusters. Cluster 3 contains the genes *dxs*, *yhfS*, *pksG*, *ispD, and mmgA* (Figure 3). On average, these genes demonstrate similarly low levels of expression in the first six perturbations (as ordered on the heat map, discussed below) and moderate to high expression in the remaining six conditions. Cluster 2 contains the genes *ispH*, *uppS*, *hepS*, *ispE*, *dxr*, and *ispF*. These genes present the opposite pattern of expression compared to cluster 1 and are highly expressed in the first six conditions compared to cluster 3. Cluster 1 contains three genes (*fni, ispG, and ispA*) that have dissimilar expression patterns compared to the other genes.

### Relationship between Isoprene Production and Terpenoid Gene Clustering

The pattern of gene expression was further examined to determine if the expression profiles of the terpenoid genes in each cluster correlate with isoprene production and whether the expression level of the cluster 2 or 3 genes might be predictive of the amount of isoprene produced. The perturbations (columns of Figure 3) are clustered based on their similarity in terpenoid gene expression. The leftmost cluster, cluster A, contains the perturbations lactic acid, acetic acid, and 0.005% and 0.02% H_2_0_2._ Given that these conditions are the lowest and highest producers of isoprene (acetic acid and H_2_0_2,_ respectively, Figure 1B), it is surprising that these four perturbations are similar to each other. The clustering of these conditions is dominated by the high level of expression of the genes in cluster 2 (*ispH*, *uppS*, *hepS*, *ispE*, *dxr*, and *ispF*) and low expression of the genes in cluster 3 (*dxs*, *yhfS*, *pksG*, and *ispD).* This suggests that the gene expression levels in these clusters are not proportional to the level of isoprene because high and low isoprene-producing conditions show similar expression levels.

**Figure 4:**
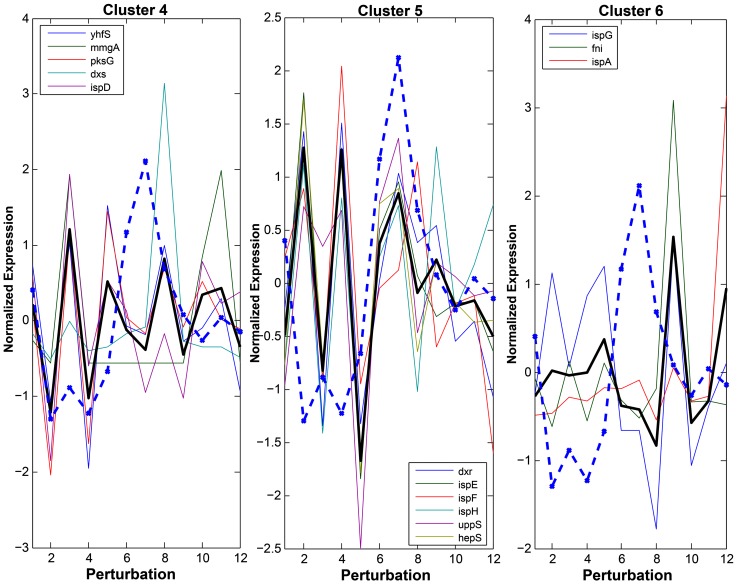
The terpenoid genes cluster into their main groups with respect to coexpression and isoprene production; covariance is dependent upon the type of perturbation. Genes were clustered using k-means. The normalized isoprene production level is represented by the dashed blue line and the mean expression for each cluster is represented by the solid black line in each cluster. The legend in each of the three panels indicates the name of the genes in each cluster. The mRNA levels are normalized (z-score) for each gene. The x-axis is representative of the perturbations as follows (1 through 12): wild type control, acetic acid, ethanol, lactic acid, indole, 0.005% H_2_O_2_, 0.02% H_2_O_2_, OX-*dxs*, OX-*fni*, DMSO, empty vector, and OX-*ispA*.

Although hierarchical clustering allowed visualization of data that successively merged similar groups of genes across conditions, this method of analysis only requires a measure of similarity between groups. We wanted to further examine the relationship between the expression of terpenoid genes and isoprene production. Therefore, we divided the terpenoid genes using k-means clustering and overlaid the isoprene production levels against the expression levels. Using this approach to represent the genes allowed us to visualize the gene expression changes with the level of isoprene produced under those perturbations. Genes in cluster 4 (Figure 4) (*yhfS, mmgA, pksG, dxs, and ispD*) show a similar expression pattern, with the exception of *mmgA*, which has very low expression levels under most conditions. The overlay of isoprene production (Figure 4, dashed blue line) shows that the expression of the genes in this cluster follows the same trend in expression as in the first five conditions (control, acetic acid, ethanol, lactic acid, and indole). Under the H_2_0_2_ conditions, in which cells produced the highest levels of isoprene, the expression levels of the genes in this cluster are low and thus do not follow the same trend as the first set of five conditions, and appear to be inversely correlated. The trend of concordant gene and isoprene expression is observed under the remaining conditions (OX-*dxs*, OX-*fni*, DMSO, empty vector, and OX-*ispA*). Analysis of the genes in cluster 5 (*dxr, ispE, ispF, ispH, uppS,* and *hepS*) revealed an inverse correlation between the gene expression level and the amount of isoprene in the first five conditions. However, under the remaining conditions, there is a positive correlation between gene expression and isoprene production. The genes in cluster 6 (*ispA, fni, ispG*) had little similarity of expression and we identified no clear relationship to isoprene production. Thus, we have identified two main clusters of terpenoid genes that covary with isoprene production. However, the nature of the correlation between these genes and isoprene production is dependent on the type of perturbation, suggesting a complicated relationship between gene expression and isoprene production.

### Correlation between Individual Terpenoid Genes and Isoprene Production

**Figure 5:**
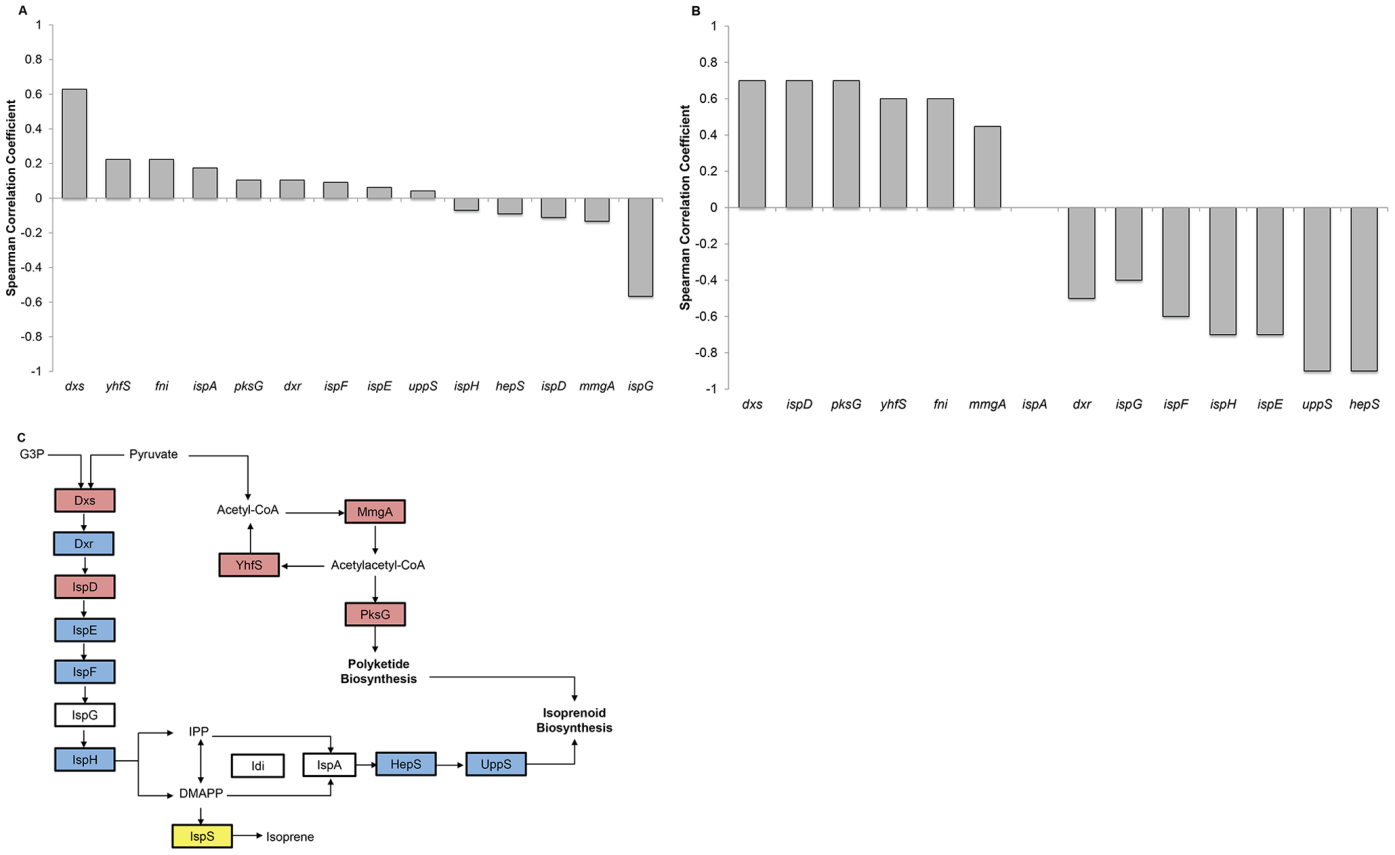
*dxs* gene expression has the strongest correlation with isoprene production. A, Spearman’s correlation coefficients were calculated between gene expression and isoprene production using all 12 perturbations; wild type control, acetic acid, ethanol, lactic acid, indole, 0.005% H_2_O_2_, 0.02% H_2_O_2_, OX-*dxs*, OX-*fni*, DMSO, empty vector, and OX-*ispA*. Refer to Figure 2A for gene names. B, Spearman’s correlation coefficients were calculated for the subset of five perturbations: wild type control, acetic acid, ethanol, lactic acid, and indole. Refer to Figure 2A for gene names. C, Genes that positively correlate with isoprene are shown in red, genes that inversely correlate with isoprene are shown in blue, genes with little correlation are not shaded, and genes with an unknown relationship are shown in yellow.

We next tested whether individual terpenoid genes show a higher correlation with isoprene production than was apparent in the previous clustering analyses. Spearman’s correlation coefficient for each gene was calculated against different subsets of perturbations; specifically, all perturbations, the first five perturbations, and all perturbations excluding H_2_0_2_. In these analyses, we found that *dxs* correlated positively with a Spearman’s correlation coefficient of 0.63 (Figure 5A), whereas *ispG* had an inverse correlation in all 12 perturbations with a Spearman’s correlation coefficient of −0.57 (Figure 5A). We did note that although *dxs* expression levels correlated with isoprene production, the range of expression values was modest, with the largest change between conditions (excluding the overexpression condition) only a 2.1 fold change (data not shown, see Discussion). The other terpenoid genes showed lower correlation to isoprene levels under these conditions. When analyzing the first five perturbations (control, acetic acid, ethanol, lactic acid, and indole), the correlation between isoprene and gene expression increased for several genes. The expression levels of *dxs*, *ispD, pksG, yhfS*, and *fni* have coefficient correlations of approximately 0.6 (Figure 5B). These genes, with the exception of *fni*, all cluster together in the hierarchical and k-means clustering (Figures 3, 4). Several genes show a strong inverse relationship (Spearman’s correlation coefficient less than −0.6) with isoprene production under the first five perturbations, specifically *ispH*, *ispE, uppS*, and *hepS* (Figure 5B). Thus, analyses of the terpenoid gene expression profiles indicate that under the acetic acid, lactic acid, ethanol, and indole conditions, there are subsets of genes that strongly correlate with isoprene production (one group positively and the other negatively) (Figure 5C).

### Genome Wide Expression Analyses and the Relationship to Isoprene Production

**Figure 6:**
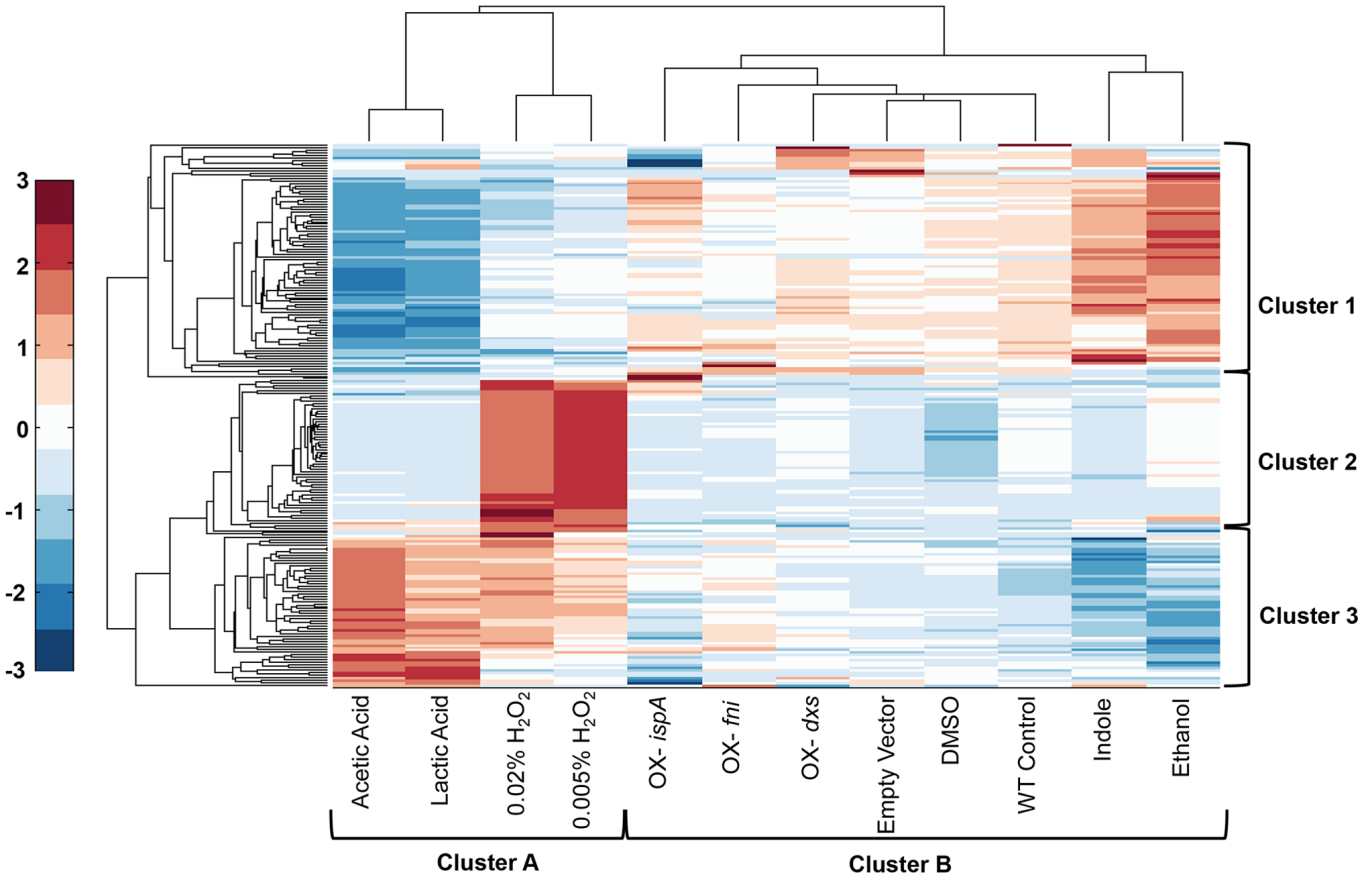
Clustering of the reduced transcriptome identifies similar expression patterns in acetic acid, lactic acid, and H_2_0_2_ perturbations. Genes and conditions were clustered using squared Euclidean distance metric. The genes in each cluster are listed in Tables S2, S3, S4.

Although the expression clusters of terpenoid genes were predictive of isoprene production under certain perturbations, we were interested in predicting isoprene levels under all the perturbations tested, thus providing a broader investigation into the mechanisms of isoprene production. Therefore, we sought to determine whether the expression levels of additional genes outside the terpenoid pathway would contribute to a model to predict isoprene production. Identification of these genes could help provide information regarding the processes that are important for isoprene production, such as pathways that regulate substrate or cofactor levels that are necessary for isoprene production. These expanded analyses included the original 14 terpenoid genes (Figure 2A) as well as an additional 199 genes selected by their differential regulation in at least one perturbation compared to the wild type control (refer to Materials and Methods). These genes were clustered by their expression levels across the 12 perturbations. Clustering the perturbations (columns) with this expanded set of genes results in clustering the H_2_0_2_ conditions with the lactic acid and acetic acid conditions (cluster C, Figure 6), the same clustering pattern observed when analyzing the terpenoid genes alone. This suggests that at the transcriptional level, the cellular response is dramatically different under these perturbations compared to any of the other conditions (cluster D, Figure 6). Because these are conditions of extremely high (H_2_0_2_) or low (acetic acid and lactic acid) isoprene production, it is surprising that the gene expression state, which represents the state of the cell (e.g., stressed, actively growing, sporulation, etc.), is dramatically different than other conditions that correspond to moderate isoprene production levels.

We investigated whether the identity of the genes that drive the clustering are informative of the differential cell state. The top cluster of 93 genes (Cluster 7, Figure 6) are expressed at much lower levels in the perturbations with H_2_0_2_ and the acidic conditions (Cluster C, Figure 6) compared to other perturbations. This group is enriched with genes involved in antibiotic metabolic processes and genes involved in polyketide biosynthesis, including *pksG* (Table S3) (GO analysis; Benjamini Hochberg *p* = 1.7×10^−7^ compared to the entire transcriptome). The H_2_0_2_ conditions contain a cluster (cluster 8) of 62 genes that were highly expressed. This group has a high proportion of genes (58%) that encode proteins predicted to be involved in prophage or phage-like elements (Table S4). This finding supports previous studies that determined these elements were involved in *B. subtilis* responses to H_2_O_2_ exposure [46], including the prophage element PBSX, indicative of initiation of sporulation and autolytic activities [47], [48]. The bottom cluster of 57 genes (Cluster 9, Figure 6) is highly expressed in H_2_0_2_ conditions, with higher expression in the lactic acid and acetic acid conditions. This group is enriched with genes involved in translation, including several 30S and 50S subunits, elongation, and translation factors (Table S5) (GO analysis; Benjamini Hochberg *p* = 7.5×10^−10^ compared to entire transcriptome). Thus, these expanded gene expression analyses demonstrate that the transcriptome of cells expressing high or low isoprene are surprisingly similar, particularly compared with the other perturbations.

### Predicting Isoprene Production Levels from Gene Expression Profiles

**Figure 7:**
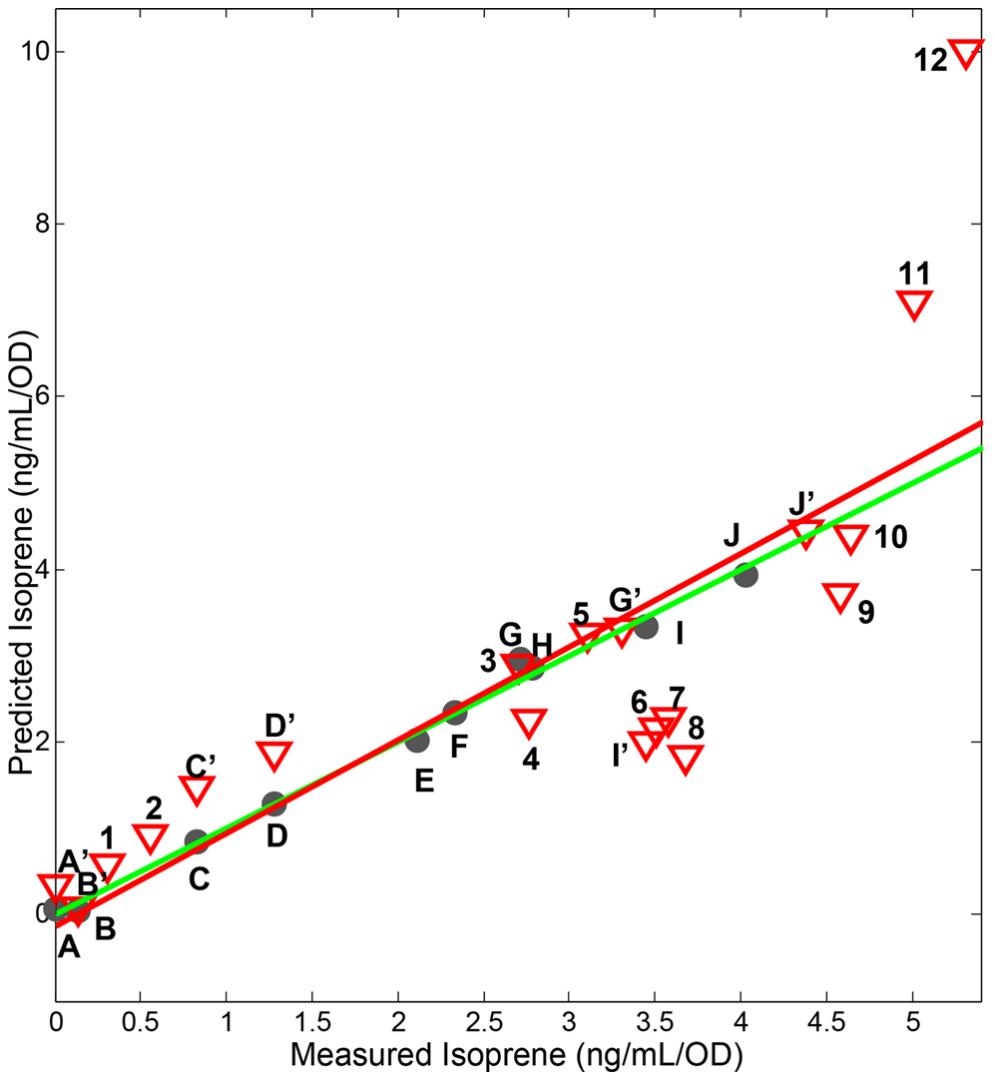
Isoprene production induced by perturbations can be predicted by a PLSR model based on a reduced transcriptome. The model was created using 213 genes and trained using ten perturbations with cross validation. The green line is the fit for the training data set; the red line is the fit for the testing data set. The R^2^ value for prediction of the test set is 0.64. Closed circles are representative of training set values that are labeled A through J; the red triangles are the test set conditions, the test set conditions that are identical to the training set conditions are labeled A’ through J’. Unique conditions in the training set are labeled numerically 1 through 12. Perturbation abbreviations: A and A’, 2% acetic acid; B and B’, 2% lactic acid; C and C’, 1% ethanol; D and D’, 0.2 mg/mL indole; E, DMSO; F, OX-*ispA*; G and G’, OX-*fni*, H, empty vector; I and I’, wild type control; J and J’, OX-*dxs*; 1, 0.2% acetic acid; 2, 0.2% lactic acid; 3, 0.02 mg/mL indole; 4, 0.1% ethanol; 5, 0.2% xylose; 6, 0.2% mannose; 7, 0.2% glucose; 8, 0.3 M NaCl; 9, OX-*dxsdxr*; 10, OX-*dxsfni*; 11, 0.005% H_2_O_2_; 12, 0.02% H_2_O_2_.

Based on the clustering data, we hypothesized that rather than a simple linear relationship between groups of genes to predict isoprene production, a more accurate prediction could be made by examining the relative importance of different gene expression levels on isoprene production. Therefore, we created a model to predict isoprene production using partial least squares regression (PLSR) analyses [49]. This model was created using the 213 genes described above to evaluate the significance of each gene for determining the level of isoprene produced. Our initial PLSR model was created with 12 perturbations constituting the training set. This model was tested against RNA profiles from an independent experiment of 19 perturbations, some of which are not present in the training set (Table 1). It resulted in a high predictive capacity for isoprene production (prediction fit of R^2^ = 0.69). A model created after randomizing the isoprene levels yielded a poor model (R^2^ = 0.25). Because the extremely high level of isoprene produced in the H_2_0_2_ perturbations is likely to dominate the predictions of the model, a reduced training set was used that did not include the H_2_0_2_ perturbations. The final model was created with this reduced training set of 10 perturbations, using three latent variables (LV), which together captured 99.4% of the variance in the isoprene levels. When the model was applied to the test set, the model accurately predicted the isoprene production with prediction fit of R^2^ = 0.64 (Figure 7). The level of isoprene produced in the H_2_0_2_ perturbations, although not in the training data, was accurately predicted to be the highest isoprene producer; however, the model predicted a lower level of isoprene than was observed in the experiment. These results demonstrate that the set of 213 genes includes the necessary information to predict the relative amount of isoprene produce in novel cellular perturbations.

**Table 1:** Concentration of isoprene produced in*B. subtilis* under different environmental and genetic perturbations.

Perturbation	Isoprene Concentration (ng/mL/OD_600_)
Wild Type Control	3.64±0.49
0.2% Acetic Acid	0.13±0.16
2% Acetic Acid	0.00±0.00
0.1% Ethanol	2.69±0.12
1% Ethanol	1.06±0.21
0.2% Lactic Acid	0.34±0.20
2% Lactic Acid	0.10±0.04
0.02 mg/mL Indole	2.54±0.21
0.2 mg/mL Indole	1.21±0.10
0.005% H_2_O_2_	4.82±0.38
0.02% H_2_O_2_	5.43±0.16
0.3 M NaCl	3.87±0.22
0.2% Glucose	3.69±0.15
0.2% Mannose	3.81±0.42
0.2% Xylose	3.55±0.62
OX-*dxs*	4.59±0.55
OX-*dxsdxr*	4.12±0.44
OX-*dxsfni*	4.23±0.59
OX-*fni*	3.14±0.24

“OX” represents genetic perturbations in which the listed gene(s) was overexpressed.

**Figure 8:**
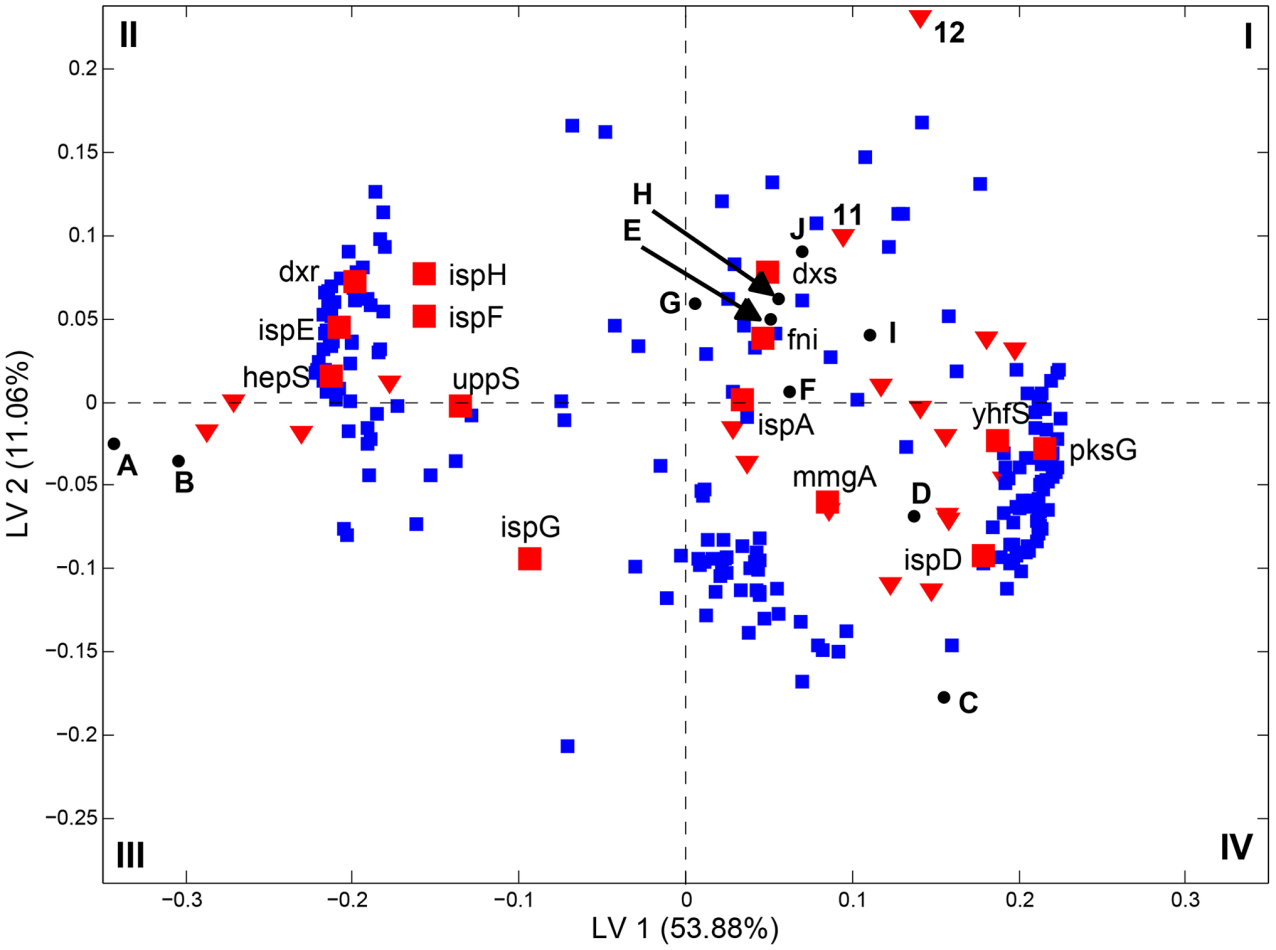
Gene expression levels are predictive of isoprene production in a PLSR model. A total of 213 genes were used in the analyses. Genes (squares), terpenoid genes (red squares), training set perturbations (red triangles), and test set perturbations (closed circles) are indicated in the plot. LV1 and LV2 indicate the latent variable values for each axis, with the percent variance in isoprene captured by the model indicated in parentheses. Perturbation abbreviations for the training set are labeled A through J and two perturbations for the testing data set are labeled as 11 and 12. Abbreviations: A, 2% acetic acid; B, 2% lactic acid; C, 1% ethanol; D, 0.2 mg/mL indole; E, DMSO; F, OX-*ispA*; G, OX-*fni*, H, empty vector; I, wild type control; J, OX-*dxs*; 11, 0.005% H_2_O_2_; 12, 0.02% H_2_O_2_.

To gain a clear understanding of how our model predicts isoprene production levels, we examined how the gene expression variables of the model influenced the prediction. The PLSR model is created by assigning weights to each variable; in this case, individual genes. The locations of each gene are indicated by its position (technically, its projection) in the biplot (Figure 8, Table S6), and the weights of the responses are indicated for each of the perturbations analyzed in the test and training dataset by their position in the biplot. This plot reveals that the predictive model assigns high positive values for the H_2_0_2_ and OX-*dxs* perturbations, which produce the highest isoprene levels. The genes that have the highest weights in LV1 (x-axis of Figure 8) and LV2 (y-axis of Figure 8) are those that have the greatest influence in the model to predict high isoprene production. Of the terpenoid genes, only *dxs* is located in this area of the biplot (Quadrant I), supporting its role as a key regulator of isoprene production. The genes *dxr*, *ispE*, *hepS*, *ispH*, and *ispF* are located along the negative region of LV1 and along the axis of LV2 (Quadrant II). This is consistent with a negative influence on isoprene production because this is proximal to the region of the biplot with perturbations for low isoprene production. The dense cluster of genes near *dxr* is enriched for genes involved in translation, suggesting that increased expression of these genes is predictive of low isoprene production (Figure 8, Table S6). The cluster in Quadrant IV (grouped near the terpenoid genes *pksG*, *ispD*, and *yhfS*) is enriched for genes involved in antibiotic metabolism as well as genes involved in amino acid metabolism (Figure 8, Table S6). This model indicates that the level of isoprene produced is best predicted by considering the expression of not only terpenoid genes, but also using genes not normally associated with this pathway.

## Discussion

The production of isoprene and isoprenoids from bacteria has great potential for industrial and medical applications. However, the lack of a complete understanding of the regulation and constraints inherent to the terpenoid pathway discourages practical applications. To address these limitations, we sought to identify the relationship between gene expression and isoprene production in *B. subtilis* and found that the terpenoid genes fall into two main groups: one group has a strong positive correlation with isoprene production, and the other has a strong negative correlation with isoprene production. The expression levels of these genes, along with a subset of genes representative of other pathways, were predictive of isoprene levels in novel perturbations. These data suggest that further investigation into the influence of other metabolic pathways will support efforts to modulate isoprene production in model organisms.

The first step in the DXP pathway, condensation of pyruvate and glyceraldehyde-3-phosphate, is mediated by Dxs and is the rate-limiting step for isoprene (and isoprenoid production) in plants and bacteria [9], [45]. Here, we show that *dxs* overexpression drives isoprene production and that its expression correlates with isoprene production in nearly all perturbations. Accordingly, the expression level of *dxs* strongly influences the PLSR model that accurately predicts the amount of isoprene produced, in contrast with other terpenoid genes that either moderately or negatively correlate to isoprene according to the model (Figures 7, 8). Dxs is known to be negatively regulated by RelA during starvation, known as the stringent response, a response that modulates ∼142 genes [50]. Consistently, our analyses reveal that *relA* expression negatively correlated with *dxs* expression in low isoprene conditions (data not shown). The *dxs* gene is predicted to be part of a four gene operon including *recN, ahrC, and yqxC* [51]. However, the role of these other gene products in the terpenoid pathway is unknown. Surprisingly, the magnitude of *dxs* regulation across conditions is modest (∼2 fold), suggesting a tight level of regulation that is consistent with its key regulatory role and strengthens the notion that *dxs* overexpression is required to ensure maximal isoprene production.

Three other terpenoid genes, *ispD*, *pksG*, and *yhfS*, also positively correlate with isoprene production. However, this correlation is strongest in a subset of conditions (wild type control, acetic acid, lactic acid, ethanol, and indole). The *ispD* gene is part of a *sigB*-dependent general stress response [52], but little has been reported regarding its expression. *pksG* and *yhfS* are part of a mevalonate pathway that is annotated as a dead-end pathway in *B. subtilis* [22], [23]. The coexpression of these genes with *dxs* and *ispD* is unexpected and suggests that these genes may have a role in regulating isoprene production, consistent with a reported link between polyketide biosynthesis and the isoprenoid synthesis [53]. In our model, *pksG* and *yhfS* have weights similar to genes involved in antibiotic metabolism, which includes several other *pksX* genes [51], [52]. Further work is required to determine the influence of the *pksG* and *yhfS* genes, and whether the mevalonate pathway in *B. subtilis* is indeed a dead-end pathway. Additionally, our analyses of coexpressed gene clusters in 104 environmental conditions (data not shown, analysis of data from [54]) demonstrated that the 14 terpenoid genes do not share any common clusters with one another (with the exception of two large clusters of 546 and 1383 genes that contained multiple terpenoid genes). Further experimental analyses and mining of publically available datasets and upstream coding regions should help extend our understanding of coregulation of the terpenoid genes. Additionally, further perturbing the nutritional and environmental constituents will likely yield important information regarding isoprene regulation.

The expression level of a second subset of genes (*dxr*, *ispE*, *ispF*, *ispH*, *uppS*, and *hepS*) was inversely correlated with isoprene in the first five conditions (control, acetic acid, lactic acid, ethanol, and indole). UppS and HepS are past the branch point of the DXP pathway and their activity is necessary to produce larger terpenoids downstream of GPP and FPP. In the absence of these enzymes, substantial amounts of these four molecules might reach toxic levels [11]. Thus, it is likely that an increase in the expression of these six genes is indicative of a shift from isoprene production to GPP/FPP production.

The *ispA* gene had limited correlation with isoprene production, but overexpression of *ispA* decreased isoprene production, indicating that diversion of the prenyl diphosphates away from isoprene synthesis to produce GPP and FPP directly affects IspS activity. The molecular cues to explain this possible phenomenon await further investigation. The inverse correlation of *dxr*, *ispE*, *ispF*, and *ispH* is more difficult to explain because these enzymes are immediately downstream of Dxs and one would intuitively predict that their expression patterns would be similar to *dxs*. Of note, the expression of *ccpA*, a transcription factor that regulates many genes involved in carbon metabolism [55], is also inversely correlated with isoprene production (data not shown). Because CcpA can function as a positive or negative regulator of transcription depending on the levels of coregulators, its role in terpenoid gene expression and isoprene production is likely to be complex [55].

Another terpenoid gene, *ispG*, also had limited correlation with isoprene production across the conditions. Recently, Zhou et al. [56] reported that the efflux of methylerythritol cyclodiphosphate, the substrate for IspG, is a rate limiting step in isoprenoid production in microbes. This rate-limiting step applies another regulatory constraint in the pathway at the level of *ispG*, perhaps providing an explanation for the more complicated relationship between its expression and isoprene production.

The mechanisms of regulating secondary metabolites such as isoprene are complex and are often intertwined with aspects of cell growth, central carbon metabolism, stress, and redox state. The first reaction of the DXP pathway utilizes pyruvate and glyceraldehyde-3-phosphate as substrates, so any limitation in these substrates due to changes in glucose or other nutrients would be expected to alter isoprene production. We expected that analyses of transcriptional changes would be a sufficient indicator of the metabolic states of cells because substantial evidence suggests that changes in metabolic activity are preceded by transcriptional changes [57], [58]. A recent report shows that in high glucose environments that cause overflow metabolism to produce excess acetate, transcriptional regulation of TCA and respiration genes is mediated by the transcription factor ArcA [59]. In *Populus trichocarpa*, which produces high levels of isoprene following an ultradian cycle, gene expression analyses found that *ispS* and *dxs* are strongly regulated at the transcriptional level and contain light-sensitive promoter elements, thus supporting a transcriptional level of regulation [60]. Although the members of the terpenoid pathway strongly correlate with isoprene production (Figure 5), gene expression level alone was insufficient to predict isoprene levels using a PLSR model (R^2^ = 0.33). However, the expression levels of these genes did indicate a high similarity between expression patterns between the perturbations with acetic acid, lactic acid, and H_2_0_2_ (Figure 5, cluster B, and Figure 6 cluster A). This is surprising considering that acidic conditions produce the lowest amount of isoprene and H_2_0_2_ perturbations produce the highest. However, this pattern of clustering was nearly identical when we clustered the perturbations using the entire transcriptome (4176 genes, not shown) and a smaller subset of regulated genes (213 genes, Figure 6).

The need for *B. subtilis* to balance its needs for carbon through central metabolism as well as secondary metabolites requires complex regulation of enzyme expression and activity. To utilize bacteria such as *B. subtilis* to produce secondary metabolites requires understanding how this balance is maintained and how it can be shifted with the desired effect. The high amount of isoprene production in *B. subtilis* compared to other bacteria, such as *E. coli* [7], may suggest a strategy of comparing the metabolic networks between these organisms in order to identify key regulatory steps in isoprene production. Our study shows that measurements of gene expression, with correlation at the transcriptional level, can provide insight into the production of isoprene across many simulated environmental conditions.

## Supporting Information

Table S1 Primer sequences used for PCR amplification and subcloning.
(DOCX)

Table S2 Expression levels of 213 genes used in clustering and modeling.
(XLS)

Table S3 ENTREZ ID and Gene Symbols for the genes in Cluster 7, Figure 6.
(DOCX)

Table S4 ENTREZ ID and Gene Symbols for the genes in Cluster 8, Figure 6.
(DOCX)

Table S5 ENTREZ ID and Gene Symbols for the genes in Cluster 9, Figure 6.
(DOCX)

Table S6 ENTREZ ID, Corresponding Gene, and Quadrant Location for the Genes in Figure 8.
(DOCX)

## References

[1] Gershenzon J, Dudareva N (2007) The function of terpene natural products in the natural world. Nat Chem Biol 3: 408–414. 17576428 10.1038/nchembio.2007.5

[2] Leonard E, Ajikumar PK, Thayer K, Xiao W-H, Mo JD, et al (2010) Combining metabolic and protein engineering of a terpenoid biosynthetic pathway for overproduction and selectivity control. Proceedings of the National Academy of Sciences 107: 13654–13659. 10.1073/pnas.1006138107 PMC2922259 20643967

[3] Xue J, Ahring BK (2011) Enhancing Isoprene Production by Genetic Modification of the 1-Deoxy-d-Xylulose-5-Phosphate Pathway in *Bacillus subtilis* . Applied and Environmental Microbiology 77: 2399–2405. 21296950 10.1128/AEM.02341-10 PMC3067423

[4] Connolly JD (1991) Dictionary of terpenoids. In: Hall/CRC C, editor. Dictionary of terpenoids. 2156.

[5] Sharkey TD, Yeh S, Wiberley AE, Falbel TG, Gong D, et al (2005) Evolution of the Isoprene Biosynthetic Pathway in Kudzu. Plant Physiology 137: 700–712. 15653811 10.1104/pp.104.054445 PMC1065370

[6] Rodríguez-Concepción M, Boronat A (2002) Elucidation of the Methylerythritol Phosphate Pathway for Isoprenoid Biosynthesis in Bacteria and Plastids. A Metabolic Milestone Achieved through Genomics. Plant Physiology 130: 1079–1089. 12427975 10.1104/pp.007138 PMC1540259

[7] Kuzma J, Nemecek-Marshall M, Pollock WH, Fall R (1995) Bacteria produce the volatile hydrocarbon isoprene. Curr Microbiol 30: 97–103. 7765889 10.1007/BF00294190

[8] Sivy TL, Shirk MC, Fall R (2002) Isoprene synthase activity parallels fluctuations of isoprene release during growth of *Bacillus subtilis* . Biochemical and Biophysical Research Communications 294: 71–75. 12054742 10.1016/S0006-291X(02)00435-7

[9] Julsing M, Rijpkema M, Woerdenbag H, Quax W, Kayser O (2007) Functional analysis of genes involved in the biosynthesis of isoprene in *Bacillus subtilis* . Applied Microbiology and Biotechnology 75: 1377–1384. 17458547 10.1007/s00253-007-0953-5 PMC1914294

[10] Martin VJJ, Pitera DJ, Withers ST, Newman JD, Keasling JD (2003) Engineering a mevalonate pathway in *Escherichia coli* for production of terpenoids. Nat Biotech 21: 796–802. 10.1038/nbt833 12778056

[11] Sivy TL, Fall R, Rosenstiel TN (2011) Evidence of Isoprenoid Precursor Toxicity in *Bacillus subtilis* . Bioscience, Biotechnology, and Biochemistry 75: 2376–2383. 10.1271/bbb.110572 22146731

[12] Lee SK, Chou H, Ham TS, Lee TS, Keasling JD (2008) Metabolic engineering of microorganisms for biofuels production: from bugs to synthetic biology to fuels. Current Opinion in Biotechnology 19: 556–563. 18996194 10.1016/j.copbio.2008.10.014

[13] Mathews J, Wang G (2009) Metabolic pathway engineering for enhanced biohydrogen production. International Journal of Hydrogen Energy 34: 7404–7416.

[14] Kleerebezemab M, Hols P, Hugenholtz J (2000) Lactic acid bacteria as a cell factory: rerouting of carbon metabolism in Lactococcus lactis by metabolic engineering. Enzyme and Microbial Technology 26: 840–848. 10862894 10.1016/s0141-0229(00)00180-0

[15] Farmer WR, Liao JC (2000) Improving lycopene production in *Escherichia coli* by engineering metabolic control. Nat Biotech 18: 533–537. 10.1038/75398 10802621

[16] Nuccio ML, Rhodest D, McNeil SD, Hanson AD (1999) Metabolic engineering of plants for osmotic stress resistance. Current Opinion in Plant Biology 2: 128–134. 10322193 10.1016/s1369-5266(99)80026-0

[17] Park SJ, Lee SY, Cho J, Kim TY, Lee JW, et al (2005) Global physiological understanding and metabolic engineering of microorganisms based on omics studies. Applied Microbiology and Biotechnology 68: 567–579. 16041571 10.1007/s00253-005-0081-z

[18] Stephanopoulos G, Vallino J (1991) Network rigidity and metabolic engineering in metabolite overproduction. Science 252: 1675–1681. 1904627 10.1126/science.1904627

[19] Lange BM, Rujan T, Martin W, Croteau R (2000) Isoprenoid biosynthesis: The evolution of two ancient and distinct pathways across genomes. Proceedings of the National Academy of Sciences 97: 13172–13177. 10.1073/pnas.240454797 PMC27197 11078528

[20] Kunst F, Ogasawara N, Moszer I, Albertini AM, Alloni G, et al (1997) The complete genome sequence of the Gram-positive bacterium *Bacillus subtilis* . Nature 390: 249–256. 9384377 10.1038/36786

[21] Shirk MC, Wagner WP, Fall R (2002) Isoprene Formation in *Bacillus subtilis*: A Barometer of Central Carbon Assimilation in a Bioreactor? Biotechnology Progress 18: 1109–1115. 12363365 10.1021/bp0255412

[22] Kanehisa M, Goto S, Sato Y, Furumichi M, Tanabe M (2012) KEGG for integration and interpretation of large-scale molecular data sets. Nucleic Acids Research 40: D109–D114. 22080510 10.1093/nar/gkr988 PMC3245020

[23] Kanehisa M, Goto S (2000) KEGG: Kyoto Encyclopedia of Genes and Genomes. Nucleic Acids Research 28: 27–30. 10592173 10.1093/nar/28.1.27 PMC102409

[24] Calderone CT, Kowtoniuk WE, Kelleher NL, Walsh CT, Dorrestein PC (2006) Convergence of isoprene and polyketide biosynthetic machinery: Isoprenyl-S-carrier proteins in the pksX pathway of *Bacillus subtilis* . Proceedings of the National Academy of Sciences 103: 8977–8982. 10.1073/pnas.0603148103 PMC1482551 16757561

[25] Keasling JD (2010) Manufacturing molecules through metabolic engineering. Science 330: 1355–1358. 21127247 10.1126/science.1193990

[26] Yang J, Xian M, Su S, Zhao G, Nie Q, et al (2012) Enhancing Production of Bio-Isoprene Using Hybrid MVA Pathway and Isoprene Synthase in *E. coli* . PLoS ONE 7: e33509. 22558074 10.1371/journal.pone.0033509 PMC3338741

[27] Withers ST, Gottlieb SS, Lieu B, Newman JD, Keasling JD (2007) Identification of Isopentenol Biosynthetic Genes from *Bacillus subtilis* by a Screening Method Based on Isoprenoid Precursor Toxicity. Applied and Environmental Microbiology 73: 6277–6283. 17693564 10.1128/AEM.00861-07 PMC2075014

[28] Nguyen H, Phan T, Schumann W (2007) Expression Vectors for the Rapid Purification of Recombinant Proteins in *Bacillus subtilis* . Current Microbiology 55: 89–93. 17624574 10.1007/s00284-006-0419-5

[29] Anders S, Huber W (2010) Differential expression analysis for sequence count data. Genome Biology 11: R106. 20979621 10.1186/gb-2010-11-10-r106 PMC3218662

[30] Sherman B, Huang D, Tan Q, Guo Y, Bour S, et al (2007) DAVID Knowledgebase: a gene-centered database integrating heterogeneous gene annotation resources to facilitate high-throughput gene functional analysis. BMC Bioinformatics 8: 426. 17980028 10.1186/1471-2105-8-426 PMC2186358

[31] Yanofsky C (2007) RNA-based regulation of genes of tryptophan synthesis and degradation, in bacteria. RNA 13: 1141–1154. 17601995 10.1261/rna.620507 PMC1924887

[32] Gollnick P, Babitzke P, Antson A, Yanofsky C (2005) Complexity in regulation of tryptophan biosynthesis in *Bacillus subtilis*. Annual Review of Genetics. Palo Alto: Annual Reviews. 47–68. 10.1146/annurev.genet.39.073003.093745 16285852

[33] Nakano MM, Hoffmann T, Zhu Y, Jahn D (1998) Nitrogen and Oxygen Regulation of *Bacillus subtilis* nasDEF Encoding NADH-Dependent Nitrite Reductase by TnrA and ResDE. Journal of Bacteriology 180: 5344–5350. 9765565 10.1128/jb.180.20.5344-5350.1998 PMC107582

[34] Yang ZW, Tendian SW, Carson WM, Brouillette WJ, Delucas LJ, et al (2004) Dimethyl sulfoxide at 2.5% (v/v) alters the structural cooperativity and unfolding mechanism of dimeric bacterial NAD+ synthetase. Protein Science 13: 830–841. 14978314 10.1110/ps.03330104 PMC2286739

[35] Velikova V, Fares S, Loreto F (2008) Isoprene and nitric oxide reduce damages in leaves exposed to oxidative stress. Plant, Cell & Environment 31: 1882–1894. 10.1111/j.1365-3040.2008.01893.x 18811730

[36] Loreto F, Velikova V (2001) Isoprene Produced by Leaves Protects the Photosynthetic Apparatus against Ozone Damage, Quenches Ozone Products, and Reduces Lipid Peroxidation of Cellular Membranes. Plant Physiology 127: 1781–1787. 11743121 PMC133581

[37] Sharkey TD, Yeh SS (2001) Isoprene emission from plants. Annual Review of Plant Physiology and Plant Molecular Biology 52: 407–436. 10.1146/annurev.arplant.52.1.407 11337404

[38] Loreto F, Mannozzi M, Maris C, Nascetti P, Ferranti F, et al (2001) Ozone quenching properties of isoprene and its antioxidant role in leaves. Plant Physiology 126: 993–1000. 11457950 10.1104/pp.126.3.993 PMC116456

[39] Wagner WP, Helmig D, Fall R (1999) Isoprene Biosynthesis in *Bacillus subtilis* via the Methylerythritol Phosphate Pathway. Journal of Natural Products 63: 37–40. 10.1021/np990286p 10650075

[40] Broun P, Somerville C (2001) Progress in plant metabolic engineering. Proceedings of the National Academy of Sciences 98: 8925–8927. 10.1073/pnas.171310598 PMC55347 11481460

[41] Kim S-W, Keasling JD (2001) Metabolic engineering of the nonmevalonate isopentenyl diphosphate synthesis pathway in *Escherichia coli* enhances lycopene production. Biotechnology and Bioengineering 72: 408–415. 11180061 10.1002/1097-0290(20000220)72:4<408::aid-bit1003>3.0.co;2-h

[42] Kajiwara S, Fraser PD, Kondo K, Misawa N (1997) Expression of an exogenous isopentenyl diphosphate isomerase gene enhances isoprenoid biosynthesis in *Escherichia coli* . Biochem J 324: 421–426. 9182699 10.1042/bj3240421 PMC1218447

[43] Yuan LZ, Rouvière PE, LaRossa RA, Suh W (2006) Chromosomal promoter replacement of the isoprenoid pathway for enhancing carotenoid production in *E. coli* . Metabolic Engineering 8: 79–90. 16257556 10.1016/j.ymben.2005.08.005

[44] Xiang S, Usunow G, Lange G, Busch M, Tong L (2007) Crystal Structure of 1-Deoxy-d-xylulose 5-Phosphate Synthase, a Crucial Enzyme for Isoprenoids Biosynthesis. Journal of Biological Chemistry 282: 2676–2682. 17135236 10.1074/jbc.M610235200

[45] Harker M, Bramley PM (1999) Expression of prokaryotic 1-deoxy-d-xylulose-5-phosphatases in *Escherichia coli* increases carotenoid and ubiquinone biosynthesis. FEBS Letters 448: 115–119. 10217421 10.1016/s0014-5793(99)00360-9

[46] Fein JE, Rogers HJ (1976) Autolytic enzyme-deficient mutants of *Bacillus subtilis* 168. Journal of Bacteriology 127: 1427–1442. 821929 10.1128/jb.127.3.1427-1442.1976 PMC232939

[47] Hartford OM, Dowds BCA (1992) Cloning and characterization of genes induced by hydrogen peroxide in *Bacillus subtilis* . Journal of General Microbiology 138: 2061–2068. 1479342 10.1099/00221287-138-10-2061

[48] Krogh S, O’Reilly M, Nolan N, Devine KM (1996) The phage-like element PBSX and part of the skin element, which are resident at different locations on the *Bacillus subtilis* chromosome, are highly homologous. Microbiology 142: 2031–2040. 8760915 10.1099/13500872-142-8-2031

[49] Karin JJ, Kevin AJ (2012) Modeling the latent dimensions of multivariate signaling datasets. Physical Biology 9: 045004. 22871687 10.1088/1478-3975/9/4/045004 PMC3769421

[50] Eymann C, Homuth G, Scharf C, Hecker M (2002) *Bacillus subtilis* functional genomics: global characterization of the stringent response by proteome and transcriptome analysis. Journal of Bacteriology 184: 2500–2520. 11948165 10.1128/JB.184.9.2500-2520.2002 PMC134987

[51] Price MN, Huang KH, Alm EJ, Arkin AP (2005) A novel method for accurate operon predictions in all sequenced prokaryotes. Nucleic Acids Research 33: 880–892. 15701760 10.1093/nar/gki232 PMC549399

[52] Mäder U, Schmeisky AG, Flórez LA, Stülke J (2012) SubtiWiki–a comprehensive community resource for the model organism *Bacillus subtilis* . Nucleic Acids Research 40: D1278–D1287. 22096228 10.1093/nar/gkr923 PMC3245094

[53] Butcher RA, Schroeder FC, Fischbach MA, Straight PD, Kolter R, et al (2007) The Identification of Bacillaene, the Product of the PksX Megacomplex in *Bacillus subtilis* . Proceedings of the National Academy of Sciences of the United States of America 104: 1506–1509. 17234808 10.1073/pnas.0610503104 PMC1785240

[54] Nicolas P, Mäder U, Dervyn E, Rochat T, Leduc A, et al (2012) Condition-Dependent Transcriptome Reveals High-Level Regulatory Architecture in *Bacillus subtilis* . Science 335: 1103–1106. 22383849 10.1126/science.1206848

[55] Sonenshein AL (2007) Control of key metabolic intersections in *Bacillus subtilis* . Nat Rev Micro 5: 917–927. 10.1038/nrmicro1772 17982469

[56] Zhou K, Zou R, Stephanopoulos G, Too H-P (2012) Metabolite Profiling Identified Methylerythritol Cyclodiphosphate Efflux as a Limiting Step in Microbial Isoprenoid Production. PLoS ONE 7: e47513. 23133596 10.1371/journal.pone.0047513 PMC3487848

[57] Buescher JM, Liebermeister W, Jules M, Uhr M, Muntel J, et al (2012) Global Network Reorganization During Dynamic Adaptations of *Bacillus subtilis* Metabolism. Science 335: 1099–1103. 22383848 10.1126/science.1206871

[58] Chechik G, Oh E, Rando O, Weissman J, Regev A, et al (2008) Activity motifs reveal principles of timing in transcriptional control of the yeast metabolic network. Nat Biotech 26: 1251–1259. 10.1038/nbt.1499 PMC2651818 18953355

[59] Valgepea K, Adamberg K, Nahku R, Lahtvee P-J, Arike L, et al (2010) Systems biology approach reveals that overflow metabolism of acetate in *Escherichia coli* is triggered by carbon catabolite repression of acetyl-CoA synthetase. BMC Systems Biology 4: 166. 21122111 10.1186/1752-0509-4-166 PMC3014970

[60] Wiberley AE, Donohue AR, Westphal MM, Sharkey TD (2009) Regulation of isoprene emission from poplar leaves throughout a day. Plant, Cell & Environment 32: 939–947. 10.1111/j.1365-3040.2009.01980.x 19389050

