# Supplementary material for: Coregulation of Terpenoid Pathway Genes and Prediction of Isoprene Production in Bacillus subtilis Using Transcriptomics
Source: PLoS One. 2013 Jun 19;8(6):e66104. doi: 10.1371/journal.pone.0066104 (PMC3686787; doi:10.1371/journal.pone.0066104)
Supplement: Table S1 — Primer sequences used for PCR amplification and subcloning. (DOCX) [file pone.0066104.s001.docx]

**Table S1. Primer sequences used for PCR amplification and subcloning**

| Primer^1^ | Sequence^2^ |
| --- | --- |
| *dxs*-BamHI-F | CG**GGATCC**TTGGATCTTTTATCAATACAGGAC |
| *dxs*-XbaI-R | GC**TCTAGA**TCATGATCCAATTCCTTTGTG |
| *dxr*-BamHI-F | CG**GGATCC**TTGAAAAATATTTGTCTTTTAGGAGCA |
| *dxr*-XbaI-R | GC**TCTAGA** TCA CGA ACA TAC CAC CTT ATG |
| *dxr*-SOE-F | CACAAAGGAATTGGATCATGATTGAAAAATATTTGT CTT TTAGGAGCA |
| *dxs*-SOE-R | TCATGATCCAATTCCTTTGTG |
| *ispA*-BamHI-F | CGGGATCC GTGACAAATAAATTAACGAGCTTTCTGGC |
| *ispA*-XbaI-F1 | GC**TCTAGA**TTAGTGATCTCTTGCCGCAATTA |
| *fni*-BamHI-F | CGGGATCCGTGACTCGAGCAGAACGAAAAAGA |
| *fni*-XbaI-R | GCTCTAGATTATCGCACACTATAGCTTGA |
| *fni*-SOE-F | CACAAAGGAATTGGATCATGAGTGACTCGAGCAGAACGAAAAAGA |

*^1^* F, forward primer; R, reverse primer.

*^2^* Primers were designed using published sequences available at the GenoList server (http://genodb.pasteur.fr/cgi-bin/WebObjects/GenoList). The restriction sites incorporated into primers for cloning purposes are shown in boldface and underlined. The overhangs complementary to the genes in the double expression mutants are underlined in the “SOE” primer sequences.
