# Supplementary material for: Coregulation of Terpenoid Pathway Genes and Prediction of Isoprene Production in Bacillus subtilis Using Transcriptomics
Source: PLoS One. 2013 Jun 19;8(6):e66104. doi: 10.1371/journal.pone.0066104 (PMC3686787; doi:10.1371/journal.pone.0066104)
Supplement: Table S3 — ENTREZ ID and Gene Symbols for the genes in Cluster 7, Figure 6. (DOCX) [file pone.0066104.s003.docx]

**Table S3. ENTREZ ID and Gene Symbols for the genes in Cluster 7, Figure 6**

| Entrez ID | Gene Symbol |
| --- | --- |
| 935957 | *liaR* |
| 935958 | *liaI* |
| 935967 | *liaS* |
| 935970 | *yvqJ* |
| 935981 | *nprE* |
| 936030 | *yraI* |
| 936131 | *yfkN* |
| 936168 | *ycsN* |
| 936241 | *yhcR* |
| 936355 | *yisT* |
| 936400 | *appB* |
| 936432 | *yvdQ* |
| 936433 | *xynD* |
| 936452 | *yjiB* |
| 936570 | *yrzF* |
| 936598 | *ytkL* |
| 936715 | *yvyD* |
| 936855 | *ispD* |
| 936898 | *ctaD* |
| 936958 | *ureC* |
| 937111 | *yukJ* |
| 937220 | *rocB* |
| 937291 | *vpr* |
| 937490 | *hemL* |
| 937594 | *levR* |
| 937609 | *yraJ* |
| 937742 | *yvbY* |
| 937755 | *rocD* |
| 937760 | *argI* |
| 937761 | *rocE* |
| 937981 | *ytnP* |
| 938071 | *malS* |
| 938159 | *yvfW* |
| 938268 | *glcU* |
| 938300 | *srfAD* |
| 938306 | *srfAA* |
| 938308 | *srfAC* |
| 938338 | *ycgM* |
| 938471 | *liaG* |
| 938495 | *rocC* |
| 938507 | *yrhH* |
| 938549 | *gcvPB* |
| 938609 | *dxs* |
| 938652 | *ispA* |
| 938662 | *mmgA* |
| 938670 | *bcd* |
| 938672 | *bkdAB* |
| 938687 | *yqjE* |
| 938693 | *yqjL* |
| 938696 | *yqjN* |
| 938729 | *sigF* |
| 938753 | *yeeF* |
| 938800 | *yfmA* |
| 938852 | *yuxJ* |
| 938930 | *spoIIAB* |
| 938960 | *resD* |
| 938962 | *resC* |
| 938965 | *resE* |
| 938985 | *fni* |
| 939016 | *ypiB* |
| 939289 | *clpE* |
| 939311 | *yhfS* |
| 939320 | *mtnB* |
| 939322 | *mtnD* |
| 939332 | *ogt* |
| 939402 | *yjiC* |
| 939416 | *yjoA* |
| 939417 | *rapA* |
| 939463 | *pksD* |
| 939490 | *bdhA* |
| 939500 | *pksF* |
| 939515 | *yngG* |
| 939576 | *yncM* |
| 939581 | *ymcA* |
| 939627 | *yobO* |
| 939695 | *bpr* |
| 939909 | *ctaG* |
| 939934 | *ispA* |
| 939983 | *ppsA* |
| 939990 | *dacC* |
| 939993 | *ppsB* |
| 940001 | *ggt* |
| 940024 | *gltA* |
| 940026 | *pksM* |
| 940032 | *pksL* |
| 940033 | *pksH* |
| 940043 | *pksJ* |
| 940053 | *gltD* |
| 940054 | *pksN* |
| 940096 | *pksI* |
| 940108 | *exlX* |
| 940121 | *pksG* |
| 2914189 | *antE* |
