# Supplementary material for: Coregulation of Terpenoid Pathway Genes and Prediction of Isoprene Production in Bacillus subtilis Using Transcriptomics
Source: PLoS One. 2013 Jun 19;8(6):e66104. doi: 10.1371/journal.pone.0066104 (PMC3686787; doi:10.1371/journal.pone.0066104)
Supplement: Table S4 — ENTREZ ID and Gene Symbols for the genes in Cluster 8, Figure 6. (DOCX) [file pone.0066104.s004.docx]

**Table S4. ENTREZ ID and Gene Symbols for the genes in Cluster 8, Figure 6**

| ENTREZ ID | Gene Symbol |
| --- | --- |
| 936210 | *xhlA* |
| 936237 | *xkdW* |
| 936282 | *yhaO* |
| 936464 | *xlyB* |
| 936466 | *xkdD* |
| 936470 | *xkdH* |
| 936471 | *xkdE* |
| 936474 | *xpf* |
| 936475 | *xkdT* |
| 936476 | *xkdU* |
| 936496 | *xkdJ* |
| 936500 | *xkdO* |
| 936646 | *uvrA* |
| 938148 | *ydcS* |
| 938158 | *ydcF* |
| 938161 | *xepA* |
| 938173 | *xkdQ* |
| 938181 | *xkdM* |
| 938261 | *gdh* |
| 938433 | *ybfG* |
| 938705 | *yqjW* |
| 939152 | *yomS* |
| 939158 | *yonA* |
| 939161 | *yonF* |
| 939166 | *yonK* |
| 939167 | *yonO* |
| 939170 | *yonN* |
| 939420 | *xkdB* |
| 939421 | *xkdV* |
| 939425 | *xkdI* |
| 939426 | *xtmB* |
| 939427 | *xkdR* |
| 939475 | *nrdEB* |
| 939497 | *recA* |
| 939522 | *yorW* |
| 939561 | *yosC* |
| 939732 | *xhlB* |
| 939758 | *yhaZ* |
| 939833 | *xkdF* |
| 939835 | *xtmA* |
| 939839 | *xtrA* |
| 939840 | *xkdS* |
| 939841 | *xkdC* |
| 939842 | *xkdN* |
| 939845 | *xkdG* |
| 939851 | *yorH* |
| 939869 | *xlyA* |
| 939922 | *ydcR* |
| 939941 | *xkdX* |
| 939943 | *xkdK* |
| 939946 | *xkdP* |
| 940079 | *yotJ* |
| 940114 | *yotB* |
| 8302922 | *youA* |
| 8302945 | *ykzL* |
| 8303027 | *yjzJ* |
| 8303033 | *ykzU* |
| 8303034 | *fadG* |
| 8303162 | *ykzM* |
| 8303179 | *ykzK* |
| 8303192 | *xkzB* |
| 8303196 | *xkzA* |
