# Supplementary material for: Coregulation of Terpenoid Pathway Genes and Prediction of Isoprene Production in Bacillus subtilis Using Transcriptomics
Source: PLoS One. 2013 Jun 19;8(6):e66104. doi: 10.1371/journal.pone.0066104 (PMC3686787; doi:10.1371/journal.pone.0066104)
Supplement: Table S5 — ENTREZ ID and Gene Symbols for the genes in Cluster 9, Figure 6. (DOCX) [file pone.0066104.s005.docx]

**Table S5. ENTREZ ID and Gene Symbols for the genes in Cluster 9, Figure 6**

| ENTREZ ID | Gene Symbol |
| --- | --- |
| 935952 | *rplL* |
| 935974 | *yvrH* |
| 936063 | *gatC* |
| 936153 | *rplJ* |
| 936224 | *cspB* |
| 936239 | *rplC* |
| 936368 | *rplU* |
| 936517 | *yutK* |
| 936574 | *yabR* |
| 936610 | *tig* |
| 936634 | *ispF* |
| 936645 | *rplT* |
| 936690 | *rplD* |
| 936825 | *rpsJ* |
| 936826 | *fusA* |
| 936829 | *ybxF* |
| 936950 | *ywnC* |
| 936970 | *ipk* |
| 937197 | *recR* |
| 937243 | *ytrC* |
| 937262 | *thiD* |
| 937410 | *thrS* |
| 937416 | *yxkC* |
| 937421 | *rpmI* |
| 937492 | *deaD* |
| 937528 | *tgt* |
| 937578 | *yxeC* |
| 937813 | *yqeI* |
| 937826 | *yqeM* |
| 937835 | *infC* |
| 937857 | *rimO* |
| 937900 | *ispH* |
| 937906 | *rpsR* |
| 937911 | *ssbA* |
| 937919 | *rpsF* |
| 937930 | *rnpA* |
| 937933 | *jag* |
| 937934 | *spoIIIJ* |
| 937988 | *ykhA* |
| 938243 | *ydaH* |
| 938345 | *mdr* |
| 938388 | *lmrB* |
| 938721 | *aspA* |
| 938722 | *ansA* |
| 938920 | *truA* |
| 938994 | *folE* |
| 938998 | *hepS* |
| 939051 | *cotD* |
| 939290 | *queE* |
| 939292 | *queC* |
| 939361 | *trpS* |
| 939492 | *pdhD* |
| 939599 | *rpsB* |
| 939636 | *dxr* |
| 939640 | *uppS* |
| 939669 | *yocJ* |
| 939792 | *yitL* |
