# Supplementary material for: Coregulation of Terpenoid Pathway Genes and Prediction of Isoprene Production in Bacillus subtilis Using Transcriptomics
Source: PLoS One. 2013 Jun 19;8(6):e66104. doi: 10.1371/journal.pone.0066104 (PMC3686787; doi:10.1371/journal.pone.0066104)
Supplement: Table S6 — ENTREZ ID, Corresponding Gene, and Quadrant Location for the Genes in Figure 8. (DOCX) [file pone.0066104.s006.docx]

**Table S6. ENTREZ ID, Corresponding Gene, and Quadrant Location for the Genes in Figure 8**

| Quadrant | ENTREZ ID | Gene Name |
| --- | --- | --- |
| I | 938609 | 1-deoxy-D-xylulose-5-phosphate synthase |
| I | 936474 | Positive control factor |
| I | 938268 | Glucose uptake protein glcU |
| I | 938148 | Uncharacterized protein ydcS |
| I | 939322 | Acireductone dioxygenase |
| I | 938471 | Protein liaG |
| I | 935967 | Sensor histidine kinase liaS |
| I | 938985 | Isopentenyl-diphosphate delta-isomerase |
| I | 940114 | Metallo-dependent hydrolase |
| I | 939561 | SPBc2 prophage-derived uncharacterized protein yosC |
| I | 935958 | Protein liaI |
| I | 939851 | SPBc2 prophage-derived uncharacterized protein yorH |
| I | 936432 | Uncharacterized protein yvdQ |
| I | 935957 | Transcriptional regulatory protein liaR |
| I | 939420 | Phage-like element PBSX protein xkdB |
| II | 938994 | GTP cyclohydrolase 1 |
| II | 936645 | 50S ribosomal protein L20 |
| II | 936574 | Uncharacterized protein yabR |
| II | 937988 | Uncharacterized acyl-CoA thioester hydrolase ykhA |
| II | 936063 | Glutamyl-tRNA(Gln) amidotransferase subunit C |
| II | 936690 | 50S ribosomal protein L4 |
| II | 937197 | Recombination protein recR |
| II | 937857 | Putative methylthiotransferase yqeV |
| II | 939669 | FMN-dependent NADH-azoreductase 1; FMN-dependent NADH-azoreductase 2 |
| II | 937262 | Phosphomethylpyrimidine kinase |
| II | 936239 | 50S ribosomal protein L3 |
| II | 938722 | L-asparaginase 1 |
| II | 935974 | Transcriptional regulatory protein yvrH |
| II | 936829 | Ribosome-associated protein L7Ae-like |
| II | 937528 | Queuine tRNA-ribosyltransferase |
| II | 938998 | Heptaprenyl diphosphate synthase component 1 |
| II | 939290 | BSU13740 |
| II | 938345 | Multidrug resistance protein 3 |
| II | 938158 | Uncharacterized protein ydcF |
| II | 939599 | 30S ribosomal protein S2 |
| II | 936970 | 4-diphosphocytidyl-2-C-methyl-D-erythritol kinase |
| II | 936153 | 50S ribosomal protein L10 |
| II | 936517 | Uncharacterized transporter yutK |
| II | 938721 | Aspartate ammonia-lyase |
| II | 937813 | Probable RNA-binding protein yqeI |
| II | 936825 | 30S ribosomal protein S10 |
| II | 935952 | 50S ribosomal protein L7/L12 |
| II | 939758 | BSU09810 |
| II | 936826 | Elongation factor G |
| II | 937919 | 30S ribosomal protein S6 |
| II | 937906 | 30S ribosomal protein S18 |
| II | 939051 | Spore coat protein D |
| II | 937578 | Uncharacterized protein yxeC |
| II | 938261 | Glucose 1-dehydrogenase |
| II | 937826 | Uncharacterized protein yqeM |
| II | 937930 | Ribonuclease P protein component |
| II | 937900 | 4-hydroxy-3-methylbut-2-enyl diphosphate reductase |
| II | 936950 | Uncharacterized protein ywnC |
| II | 937835 | Translation initiation factor IF-3 |
| II | 939792 | BSU11030 |
| II | 937911 | BSU40900 |
| II | 939636 | 1-deoxy-D-xylulose 5-phosphate reductoisomerase |
| II | 936224 | Cold shock protein cspB |
| II | 937492 | ATP-dependent RNA helicase dbpA |
| II | 937416 | Uncharacterized protein yxkC |
| II | 938920 | tRNA pseudouridine synthase A |
| II | 937410 | Threonyl-tRNA synthetase 1 |
| II | 938388 | Lincomycin resistance protein lmrB |
| II | 937243 | Probable ABC transporter permease ytrC |
| II | 936368 | 50S ribosomal protein L21 |
| II | 936610 | Trigger factor |
| II | 937421 | 50S ribosomal protein L35 |
| II | 939292 | Queuosine biosynthesis protein queC |
| II | 937934 | Membrane protein oxaA 1 |
| II | 936634 | 2-C-methyl-D-erythritol 2,4-cyclodiphosphate synthase |
| II | 937933 | Protein jag |
| II | 938243 | Uncharacterized protein ydaH |
| II | 939839 | Phage-like element PBSX protein xtrA |
| II | 939361 | Tryptophanyl-tRNA synthetase |
| II | 939152 | SPBc2 prophage-derived uncharacterized protein yomS |
| II | 939158 | SPBc2 prophage-derived uncharacterized protein yonA |
| II | 939492 | Dihydrolipoyl dehydrogenase |
| II | 939640 | Undecaprenyl pyrophosphate synthetase |
| III | 936210 | Protein xhlA |
| III | 939941 | Phage-like element PBSX protein xkdX |
| III | 939869 | N-acetylmuramoyl-L-alanine amidase xlyA |
| III | 939943 | Phage-like element PBSX protein xkdK |
| III | 939421 | Phage-like element PBSX protein xkdV |
| III | 936496 | Phage-like element PBSX protein xkdJ |
| III | 939833 | Phage-like element PBSX protein xkdF |
| III | 938173 | Phage-like element PBSX protein xkdQ |
| III | 939732 | Holin |
| III | 939515 | Hydroxymethylglutaryl-CoA lyase yngG |
| III | 936500 | Phage-like element PBSX protein xkdO |
| III | 936464 | N-acetylmuramoyl-L-alanine amidase xlyB |
| III | 938433 | Uncharacterized protein ybfG |
| III | 936475 | Phage-like element PBSX protein xkdT |
| III | 939845 | Phage-like element PBSX protein xkdG |
| III | 938705 | DNA polymerase IV 2 |
| III | 939427 | Phage-like element PBSX protein xkdR |
| III | 939946 | Phage-like element PBSX protein xkdP |
| III | 938662 | Acetyl-CoA acetyltransferase |
| III | 939161 | SPBc2 prophage-derived uncharacterized protein yonF |
| III | 938181 | Phage-like element PBSX protein xkdM |
| III | 939425 | Phage-like element PBSX protein xkdI |
| III | 936476 | Phage-like element PBSX protein xkdU |
| III | 936470 | Phage-like element PBSX protein xkdH |
| III | 939426 | PBSX phage terminase large subunit |
| III | 939835 | PBSX phage terminase small subunit |
| III | 939840 | Phage-like element PBSX protein xkdS |
| III | 940108 | Expansin-yoaJ |
| III | 938161 | Phage-like element PBSX protein xepA |
| III | 936471 | Phage-like element PBSX protein xkdE |
| III | 936237 | Phage-like element PBSX protein xkdW |
| III | 939475 | Ribonucleoside-diphosphate reductase nrdEB subunit alpha |
| III | 939842 | Phage-like element PBSX protein xkdN |
| IV | 940032 | Putative polyketide synthase pksL |
| IV | 936958 | Urease subunit alpha |
| IV | 936030 | Uncharacterized protein yraI |
| IV | 938729 | RNA polymerase sigma-F factor |
| IV | 936355 | Uncharacterized protein yisT |
| IV | 940024 | Glutamate synthase [NADPH] large chain |
| IV | 937760 | Arginase |
| IV | 937981 | Uncharacterized protein ytnP |
| IV | 940001 | Gamma-glutamyltranspeptidase |
| IV | 936452 | Putative cytochrome P450 yjiB |
| IV | 939934 | Major intracellular serine protease |
| IV | 937755 | Ornithine aminotransferase |
| IV | 937609 | Uncharacterized protein yraJ |
| IV | 939416 | Uncharacterized protein yjoA |
| IV | 936131 | BSU07840 |
| IV | 939332 | Methylated-DNA--protein-cysteine methyltransferase |
| IV | 939402 | Uncharacterized UDP-glucosyltransferase yjiC |
| IV | 939576 | Uncharacterized protein yncM |
| IV | 938071 | Probable NAD-dependent malic enzyme 3 |
| IV | 936433 | Arabinoxylan arabinofuranohydrolase |
| IV | 940053 | Glutamate synthase [NADPH] small chain |
| IV | 938962 | Cytochrome c biogenesis protein resC |
| IV | 938670 | Leucine dehydrogenase |
| IV | 938549 | Probable glycine dehydrogenase [decarboxylating] subunit 2 |
| IV | 936400 | Oligopeptide transport system permease protein appB |
| IV | 935981 | Bacillolysin |
| IV | 938338 | Proline dehydrogenase 2 |
| IV | 936241 | Endonuclease yhcR |
| IV | 937742 | BSU34030 |
| IV | 940033 | Putative polyketide biosynthesis enoyl-CoA hydratase homolog pksH |
| IV | 940026 | Putative polyketide synthase pksM |
| IV | 938930 | Anti-sigma F factor |
| IV | 939463 | BSU17110 |
| IV | 936570 | BSU27780 |
| IV | 939983 | Plipastatin synthetase subunit A |
| IV | 936646 | UvrABC system protein A |
| IV | 939500 | Putative polyketide beta-ketoacyl synthase |
| IV | 939909 | Protein ctaG |
| IV | 938495 | Amino-acid permease rocC |
| IV | 2914189 | Protein antE |
| IV | 938672 | 2-oxoisovalerate dehydrogenase subunit beta |
| IV | 939016 | UPF0302 protein ypiB |
| IV | 938965 | Sensor histidine kinase resE |
| IV | 936898 | Cytochrome c oxidase subunit 1 |
| IV | 938159 | BSU34040 |
| IV | 937761 | Amino-acid permease rocE |
| IV | 939990 | D-alanyl-D-alanine carboxypeptidase dacC |
| IV | 935970 | Uncharacterized MFS-type transporter yvqJ |
| IV | 939311 | Putative acetyl-CoA C-acetyltransferase yhfS |
| IV | 939695 | Bacillopeptidase F |
| IV | 939627 | pre-neck appendage protein (A) yobO |
| IV | 936715 | Uncharacterized protein yvyD |
| IV | 936855 | 2-C-methyl-D-erythritol 4-phosphate cytidylyltransferase |
| IV | 938693 | Uncharacterized protein yqjL |
| IV | 939417 | Response regulator aspartate phosphatase A |
| IV | 937490 | Glutamate-1-semialdehyde 2 |
| IV | 938960 | Transcriptional regulatory protein resD |
| IV | 940121 | Putative polyketide biosynthesis protein pksG |
| IV | 940054 | BSU17210 |
| IV | 936598 | UPF0173 metal-dependent hydrolase ytkL |
| IV | 939581 | Uncharacterized protein ymcA |
| IV | 940043 | Putative polyketide synthase pksJ |
| IV | 938852 | Uncharacterized MFS-type transporter yuxJ |
| IV | 936168 | Uncharacterized oxidoreductase ycsN |
| IV | 937594 | Transcriptional regulatory protein levR |
| IV | 940096 | Putative polyketide biosynthesis enoyl-CoA hydratase homolog pksI |
| IV | 939993 | Plipastatin synthetase subunit B |
| IV | 937111 | Hypothetical Protein yukJ |
| IV | 938696 | Uncharacterized protein yqjN |
| IV | 937291 | Minor extracellular protease vpr |
| IV | 937220 | Protein rocB |
| IV | 939490 | Acetoin Reductase bdhA |
